# Multiomics implicate gut microbiota in altered lipid and energy metabolism in Parkinson’s disease

**DOI:** 10.1038/s41531-022-00300-3

**Published:** 2022-04-11

**Authors:** Pedro A. B. Pereira, Drupad K. Trivedi, Justin Silverman, Ilhan Cem Duru, Lars Paulin, Petri Auvinen, Filip Scheperjans

**Affiliations:** 1 Department of Neurology, Helsinki University Hospital, and Clinicum, University of Helsinki, Haartmaninkatu 4, 00290 Helsinki, Finland; 2 Institute of Biotechnology, DNA Sequencing and Genomics Laboratory, University of Helsinki, Viikinkaari 5D, 00014 Helsinki, Finland; 3 Manchester Institute of Biotechnology, The University of Manchester, 131 Princess Street, Manchester, M1 7DN UK; 4 College of Information Science and Technology, Department of Statistics, and Institute for Computational and Data Science, Penn State University, University Park, PA USA; 5 Department of Medicine, Penn State University, Hershey, PA USA

**Keywords:** Parkinson's disease, Metabolomics, Next-generation sequencing

## Abstract

We aimed to investigate the link between serum metabolites, gut bacterial community composition, and clinical variables in Parkinson’s disease (PD) and healthy control subjects (HC). A total of 124 subjects were part of the study (63 PD patients and 61 HC subjects). 139 metabolite features were found to be predictive between the PD and Control groups. No associations were found between metabolite features and within-PD clinical variables. The results suggest alterations in serum metabolite profiles in PD, and the results of correlation analysis between metabolite features and microbiota suggest that several bacterial taxa are associated with altered lipid and energy metabolism in PD.

## Introduction

Parkinson’ disease (PD) is the second most common neurodegenerative disorder and is associated with prominent gastrointestinal pathophysiological changes and symptoms^1^. In the gut of PD patients, there are signs of low-grade inflammation, increased permeability, and bacterial invasion, all of which may predispose to overexpression and accumulation of alpha-synuclein that subsequently may spread to the brain in a prion-like fashion. Indeed, recent research suggests that early gastrointestinal involvement may be a key determinant of PD subtypes and that in a significant group of patients the origin of PD may lie in the gut. Gut microbiota impact brain health through multiple pathways, including production of neuroactive metabolites and neurotransmitters, but also through interactions with the immune system and potentially by excreting aggregation-prone proteins. Compositional alterations of gut microbiota in PD have been robustly demonstrated across multiple cohorts^2,3^ and have been linked to motor- and non-motor symptoms as well as progression of the disease. However, the functional implications of these changes regarding microbiota-host interactions and PD pathology and progression are still poorly understood. Alterations of faecal and serum/plasma metabolites and inflammatory markers have been described in relation to gut microbiota^4–6^, but except for reproducible findings of reduced faecal short-chain fatty acid (SCFA) levels in PD^7^, shortlisting of relevant metabolites and pathways has been challenging and inconsistent. Multiomics analyses have linked faecal microbiota abundances to alterations of amino acid metabolites^4^, lipids, sulphur metabolism, bile acids^8^, and SCFAs^9^ in serum/plasma, and alterations of lipids, vitamins, amino acids, SCFAs, and other organic compounds in faeces of PD patients^10–12^. Thus, more research is needed to better understand how an altered microbial composition and metabolic activity may impact PD.

The Helsinki cohort has so far been analysed for microbiome correlations with clinical features^2,13^ and disease progression^14^. We have also studied the oral and nasal microbe communities^15^. Recently, the immune response and fecal SFCA levels were studied among the same individuals^6^. The current study was primarily designed to investigate, as broadly as possible, the existence of possible links between gut bacteria and metabolite features, using a data-driven, hypothesis-generating approach (as opposed to hypothesis-testing approach), using untargeted serum metabolomics, 16S rRNA bacterial marker gene data, and clinical symptoms in PD as compared to healthy control subjects (HC). A total of 124 subjects were part of the study, divided into 63 PD patients and 61 HC subjects^14^ (see supplementary file “Supplementary population data table” for details). The serum samples were collected as close to the collection timepoint of the stool samples as practically possible, with the following average difference between stool and serum collection (days mean ± SD): PD (1.27 ± 1.42) and HC (0.69 ± 2.20).

## Results

### Metabolome analysis

Data-driven metabolomics profiling of serum samples was undertaken, identifying a total of 7585 metabolite features. Support Vector Machines (SVM) using RBF kernel showed 81% classification accuracy using GC-MS metabolic profiles, and 77% and 72% classification accuracy was achieved using LC-MS positive and negative mode ionisation data, respectively (Table 1).

**Table 1:** Confusion matrices.

		Predicted control	Predicted PD
GC-MS CCR = 81%	Actual control	83%	17%
	Actual PD	20%	80%
LC-MS Positive Mode CCR = 77%	Actual control	75%	25%
	Actual PD	21%	79%
LC-MS Negative Mode CCR = 72%	Actual control	72%	28%
	Actual PD	28%	72%

Confusion matrices for (a) LC-MS positive mode data, (b) LC-MS negative mode data, (c) GC-MS data. For each data, confusion matrix shows an average of 100 models tested by resampling. Each time 60% data were used as training set and 40% were used as test set. Average correct classification rate (CCR) is represented for each of the data. Upon permutation of class labels, LC-MS positive mode CCR dropped to 49%, LC-MS negative mode CCR dropped to 47% and GC-MS CCR dropped to 47%.

Selection of predictive metabolite features between Controls and PD subjects was carried out using SVM recursive feature elimination to select the top 10% of features from profiling experiments. The metabolite features that contributed the most to the classification of PD and control samples in the SVM model, selected via SVM-RFE, were called “key predictive” metabolites and the same terminology will be used throughout this paper. A total of 139 features (i.e. metabolites) were selected: 101 features from LC-MS data and 38 features from GC-MS data (Table 2). Pathway enrichment analysis using all the 7585 metabolomics features revealed significant changes in carnitine shuttle, vitamin E metabolism, glycerophospholipids, sphingolipids, fatty acids and aminoacyl-tRNA biosynthesis amongst 20 perturbed pathways (Table 3). These features were also putatively annotated based on accurate mass match at 5ppm using human metabolome database (HMDB v.4)^16^ and LipidMaps^17^ following Metabolite Standards Initiative (MSI)^18^ at Level 3. Age, gender, BMI, dietary components, and PD-related clinical variables like medications did not show any evidence of biologically meaningful effects on the selected 139 metabolite features, with the possible exception of a very minor effect from COMT-inhibitors (see “Supplementary Table 7 (MODELS).xlsx” and the ‘Methods’ section for details). Within the PD group, no association was established between clinical variables and all 7585 metabolite features after adjusting for time since motor symptom onset, age at sampling, and other known clinical covariates (see also “Supplementary Table 7 (MODELS).xlsx” and the ‘Methods’ section for details). All 7585 metabolite features were used for this within-PD analysis because irrespective of their lack of predictive potential for PD (unlike the selected 139), some could still have been associated with those clinical variables that are of interest only within the PD group.

**Table 2:** Key predictive metabolite features.

Peak number	Putative ID	Metabolite feature class	Avg P/C foldchange
349	GalCer(d18:1/23:0);GlcCer(d18:1/23:0)	Sphingolipid	2.314
458	20:2-Glc-Campesterol	Sterol lipid	2.035
1303	MGDG(20:5(5Z,8Z,11Z,14Z,17Z)/18:3(9Z,12Z,15Z))	Glycerolipid	2.024
264	6-Keto-decanoylcarnitine	Fatty acyls	1.979
199	Palmitoleic acid	Fatty acyls	1.958
793	Tetrahydroaldosterone-3-glucuronide	Steroid and derivatives	1.731
856	FAHFA(18:1(9Z)/13-O-18:0)	Fatty acyls	1.722
447	Veranisatin C	Prenol lipids	1.661
1160	5-Methyltetrahydropteroyltri-L-glutamate	Steroid and derivatives	1.624
151	PE(18:4(6Z,9Z,12Z,15Z)/18:1(9Z))	Glycerophospholipid	1.604
1005	Unknown 2	Unknown	1.586
299	Neoabietic acid	Isoprenoids	1.567
429	PC(16:0/18:1(6Z));PC(16:0/18:1(6E))	Glycerophospholipid	1.488
501	Galbanic acid	Prenol lipids	1.485
368	Deca-4,6,8-triyne-1,1,2,3-tetraol	Artificial chemical	1.403
280	Citrulline	Carboxylic acid and derivatives	1.377
191	Sphinganine-phosphate	Sphingolipid	1.356
398	Unknown 1	Unknown	1.354
75	N-stearoyl tyrosine	Carboxylic acid and derivatives	1.346
32	(-)-Jolkinol B	Chemical	1.336
320	11-cis-Dehydroretinal;all-trans-Dehydroretinal	Prenol lipids	1.319
144	1alpha,24,25,28-tetrahydroxyergocalciferol	Vitamin D2 derivative	1.290
204	Glycerol	Sugar alchohol	1.289
362	PC(22:4(7Z,10Z,13Z,16Z)/0:0)	Glycerophospholipid	1.274
70	(3S,5R,6S,7E,9x)-7-Megastigmene-3,6,9-triol 9-glucoside	Fatty acyl glycoside	1.263
369	PE-Cer(d15:2(4E,6E)/22:0(2OH))	Glycerophospholipid	1.255
415	Estrone, 16alpha-hydroxy-	Steroid and derivatives	1.253
1201	PC(P-16:0/18:4(6Z,9Z,12Z,15Z))	Glycerophospholipid	1.248
1137	PE(16:0/P-18:1(11Z))	Glycerophospholipid	1.247
461	Sphingosine-1-phosphate;Sphingosine 1-phosphate	Phosphosphingolipids	1.245
386	Heptadecane, n-	Alkane	1.242
1474	PI-Cer(t20:0/22:0(2OH))	Glycerophospholipid	1.242
170	PA(P-18:0/17:2(9Z,12Z))	Glycerophospholipid	1.230
36	PG(16:1(9Z)/22:4(7Z,10Z,13Z,16Z))	Glycerophospholipid	1.229
792	PS(19:0/0:0)	Glycerophospholipid	1.219
439	PI(16:0/20:1(11Z))	Glycerophospholipid	1.209
370	3-octadecylenic acid	Fatty acyls	1.201
518	PC(18:4(6Z,9Z,12Z,15Z)/18:1(11Z))	Glycerophospholipid	1.195
67	4-O-alpha-Cadinylangolensin	Flavonoids	1.186
430	PC(P-20:0/18:3(6Z,9Z,12Z))	Glycerophospholipid	1.185
406	SM(d16:1/22:0)	Sphingolipid	1.171
393	Propionylcarnitine	Fatty acyls	1.164
420	PC(16:1(9Z)/0:0);PC(16:1(9E)/0:0)	Glycerophospholipid	1.163
47	PE(18:4(6Z,9Z,12Z,15Z)/15:1(9Z))	Glycerophospholipid	1.129
463	PC(P-16:0/20:3(8Z,11Z,14Z))	Glycerophospholipid	1.100
243	PS(20:3(8Z,11Z,14Z)/0:0)	Glycerophospholipid	1.097
414	PA(O-16:0/21:0)	Glycerophospholipid	1.097
423	PC(P-20:0/18:2(9Z,12Z))	Glycerophospholipid	1.072
497	SM(d17:1/24:1)	Sphingolipid	1.056
1339	PE(20:2(11Z,14Z)/22:5(4Z,7Z,10Z,13Z,16Z))	Glycerophospholipid	1.056
509	PI(O-16:0/13:0)	Glycerophospholipid	1.049
511	PC(18:3(9Z,12Z,15Z)/0:0)	Glycerophospholipid	1.045
21	2-amino-2-deoxy-glucose	Glucose derivative	1.043
500	Butyrylcarnitine	Fatty acyls	1.042
502	PE(P-18:0/20:5(5Z,8Z,11Z,14Z,17Z))	Glycerophospholipid	1.038
160	Methionine, N-formyl-	Amino acid derivative	1.036
499	3-Deoxyvitamin D3	Sterol lipid	1.021
161	Maltotriose	Oligosaccharides	1.014
1338	PG(P-20:0/20:1(11Z))	Glycerophospholipid	0.999
314	Proline	Carboxylic acid and derivatives	0.997
92	Alanine, beta-	Carboxylic acid and derivatives	0.996
459	PC(P-18:0/20:5(5Z,8Z,11Z,14Z,17Z))	Glycerophospholipid	0.984
170	Lactic acid, 3-imidazole-	Azoles	0.975
467	PE-Cer(d15:1(4E)/18:0)	Glycerophospholipid	0.974
1434	PI(15:0/22:0)	Glycerophospholipid	0.959
348	Cysteine, N-acetyl-	Drug	0.955
68	3-Methyl-2-oxopentanoic-acid	Neurotoxin	0.954
385	Proline	Carboxylic acid and derivatives	0.950
295	Proline, 4-hydroxy-, trans-	Carboxylic acid and derivatives	0.928
485	SM(d18:1/21:0)	Sphingolipid	0.928
1283	PE(20:4(8Z,11Z,14Z,17Z)/20:4(8Z,11Z,14Z,17Z))	Glycerophospholipid	0.925
1391	PS(19:0/22:6(4Z,7Z,10Z,13Z,16Z,19Z))	Glycerophospholipid	0.915
137	Tridecane, n-	Alkane	0.913
29	Glucose, 2-amino-2-deoxy-	Glucose derivative	0.912
498	SM(d18:2/21:0)	Sphingolipid	0.903
343	Glycine, 2-phenyl-	Carboxylic acid and derivatives	0.899
159	Serine	Amino acid	0.898
367	PC(14:0/22:6(4Z,7Z,10Z,13Z,16Z,19Z))	Glycerophospholipid	0.896
65	Dodecane	Alkane	0.896
420	Tartronic acid	Dicarboxylic acid	0.893
11	Dodecane	Alkane	0.892
109	Hydantoin, 5-methyl-	Allantoin metabolite	0.892
47	n-tricosane	Alkane	0.888
845	2-methylbacteriohopane-32,33,34,35-tetrol	Prenol lipids	0.886
247	Proline, 4-hydroxy-, trans-	Carboxylic acid and derivatives	0.885
474	Acevaltrate	Carboxylic acid	0.885
227	Pentadecane, n-	Alkane	0.883
355	Glyceric acid	Sugar acids and derivatives	0.876
126	Methionine	Amino acid	0.871
267	Aniline, 3,4-dimethyl-	Xylidine isomer	0.864
364	Decane, n-	Alkane	0.859
490	PA(O-20:0/13:0)	Glycerophospholipid	0.857
443	GlcCer(d18:1(8Z)/21:0(2OH[R]));GlcCer(d18:1(8E)/21:0(2OH[R]))	Sphingolipid	0.854
278	3-demethylubiquinone-9	Prenol lipids	0.852
323	3,3-Dibromo-2-n-hexylacrylic acid	Fatty acyls	0.852
327	Anandamide (20:5, n-3)	Fatty acid amide	0.845
384	Tetradecane, n-	Alkane	0.845
134	Unknown 4	Unknown	0.843
307	Urea	Organic acids and derivatives	0.842
387	Benzaldehyde	Benzoids	0.827
229	3,4-dimethyl-5-carboxyethyl-2-furanpentanoic acid	Furanoic fatty acids	0.826
50	Pyroglutamic acid	Carboxylic acid and derivatives	0.816
433	25-hydroxy-1alpha-hydroxymethyl-23,23,24,24-tetradehydrocholecalciferol	Vitamin D metabolite	0.815
379	Galactose, 2-amino-2-deoxy-, D-	Glucose derivative	0.800
1072	OKHdiA-PS	Chemical	0.797
360	Heptadecane, n-	Alkane	0.769
226	Norvaline, DL-	Carboxylic acid and derivatives	0.765
130	7,3’-Dihydroxy-4’-methoxy-8-methylflavan	Flavonoids	0.752
188	Unknown 3	Unknown	0.751
418	Sorbitan stearate	Sorbitol derivative	0.738
363	3-carboxy-4-methyl-5-pentyl-2-furanpropanoic acid	Furanoic fatty acids	0.703
287	Fuconic acid	Chemical	0.690
361	3,4-dimethyl-5-carboxyethyl-2-furanhexanoic acid	Furanoic fatty acids	0.690
399	PE(O-20:0/22:6(4Z,7Z,10Z,13Z,16Z,19Z))	Glycerophospholipid	0.688
880	DG(15:0/18:4(6Z,9Z,12Z,15Z)/0:0)	Fatty acyls	0.684
390	Fuconic acid	Chemical	0.669
153	Withaperuvin B	Steroid and derivatives	0.655
1044	OHOHA-PS	Chemical	0.640
9	OHOHA-PS	Chemical	0.638
337	C17 sphingosine-1-phosphocholine	Sphingolipid	0.637
201	Butenylcarnitine	Fatty acyls	0.636
274	(20S,24R)-20-fluoro-1alpha,24-dihydroxy-26,27-cyclovitamin D3	Chemical	0.634
311	Sphingofungin A	Antifungal	0.633
295	Glycoursodeoxycholic acid	Steroid and derivatives	0.610
177	Glutarylcarnitine	Fatty acyls	0.595
204	2,3-epoxyphylloquinone	Vitamin K derivative	0.572
348	Fuconic acid	Chemical	0.560
276	SM(d18:0/24:0)	Sphingolipid	0.542
263	Epigallocatechin 3-O-caffeate	Epigallocatechins	0.536
115	Hydroxybutyrylcarnitine	Fatty acyls	0.522
5	Palmitoleamide	Fatty amide	0.519
365	N-trans-Feruloyloctopamine	Cinnamic acids and derivatives	0.515
313	iodovulone I	Chemical	0.485
321	N2,N2-Dimethylguanosine	Purine nucleosides	0.353
20	Rubraflavone D	Flvonoids	0.348
24	Cycloheterophyllin	Pyranoflavonoids	0.333
331	cholesterol sulfate	Steroid and derivatives	0.328
13	Leukotriene D5	Organooxygen compounds	0.208
407	PE(20:2(11Z,14Z)/0:0)	Glycerophospholipid	0.105

Key predictive metabolite features between PD and Controls, organized in descending order of effect size. These top 10% metabolite features were selected after ranking them for their predictive power to distinguish between PD and HC. See ‘Methods’ section for details. The *m/z* features annotated as ‘Unknown’ had no accurate mass match or spectra match when compared to the library during database search. Mass spectra for these can be found in the Supplementary files as ”Supplementary Figure—Unknown X spectra”, with X corresponding to the respective unknown features 1, 2, 3, and 4 in the table.

**Table 3:** Results from pathway analysis.

Analytical Platform	Pathway name	Metabolite overlap	Pathway size	Adjusted *p*-value
LC-MS (pos mode)	Carnitine shuttle	18	27	0.00934
	Vitamin E metabolism	18	34	0.01417
	Glycosphingolipid metabolism	15	28	0.01585
	N-Glycan Degradation	5	6	0.01624
	Porphyrin metabolism	13	25	0.0209
	Glycerophospholipid metabolism	15	31	0.02649
	Saturated fatty acids beta-oxidation	8	15	0.03312
	Linoleate metabolism	9	18	0.03892
	Squalene and cholesterol biosynthesis	17	39	0.04848
LC-MS (neg mode)	De novo fatty acid biosynthesis	13	18	0.00192
	Fatty acid activation	12	17	0.00209
	Hexose phosphorylation	6	7	0.00292
	Glycosphingolipid metabolism	10	16	0.00384
	Caffeine metabolism	6	10	0.01308
	Phosphatidylinositol phosphate metabolism	4	6	0.02081
	Fructose and mannose metabolism	4	6	0.02081
	Fatty Acid Metabolism	4	6	0.02081
	Starch and Sucrose Metabolism	3	4	0.03001
	Glycerophospholipid metabolism	10	22	0.03784
GC-MS	Aminoacyl-tRNA biosynthesis	6	48	0.00011601
	Pantothenate and CoA biosynthesis	3	19	0.0037957
	Valine, leucine and isoleucine biosynthesis	2	8	0.007673
	Phenylalanine metabolism	2	10	0.012069

Results from pathway analysis for LC-MS and GC-MS data. Metabolite overlap shows the number of metabolites that overlap on the total metabolites on pathway indicated by pathway size. The *p*-values were adjusted for multiple comparisons as implemented within the Mummichog algorithm, as a penalisation process that takes into account the Cumulative Distribution Function (CDF) and the Expression Analysis Systematic Explorer (EASE).

### Metabolome-microbiome correlation analysis

Correlation analysis between the selected 139 metabolite features and bacterial taxa at genus, family, and phylum levels was performed separately for the PD and Control groups to facilitate their contrast (Supplementary Tables 1–6). All results’ tables have been curated to obtain the best possible putative identification of the metabolite peak IDs at MSI level 3 identification level. Tables 2 and 5, as well as the two genus-level supplementary tables (Supplementary Tables 1 and 2) contain a metabolite “class” identification column (see ‘Methods’ section for details). All Supplementary Tables contain all identified taxa/metabolite correlation pairs (using the selected 139 metabolite features and all bacterial abundance data) that show posterior mean correlation values at or above 0.3 and at a 95% “confidence level” (see ‘Methods’ section for more details).

**Fig. 1:**
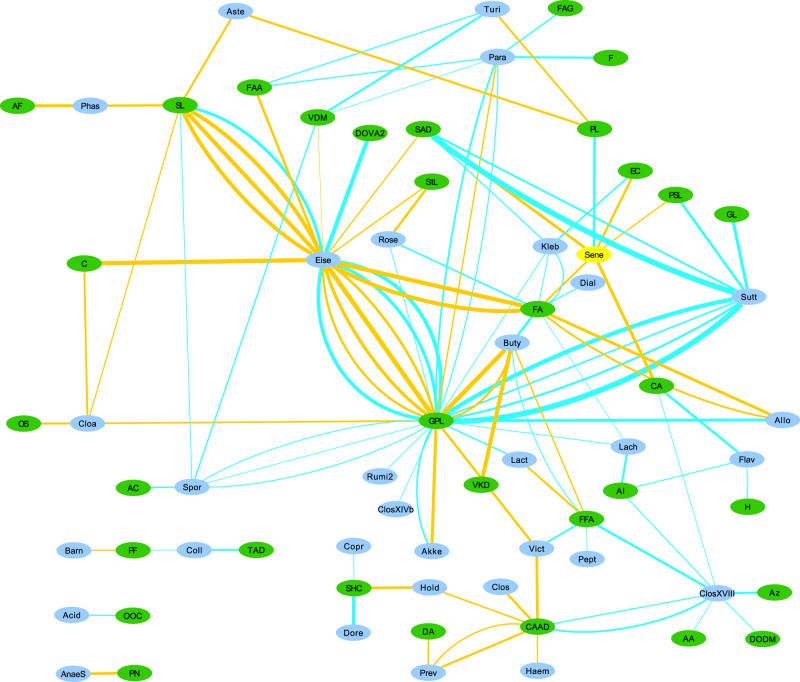
Network of within-PD correlations. Network of within-PD correlations between bacterial genera and metabolite classes. Supplementary Tables 1 and 2 contain correlations with bins composed of more than one genus (or higher taxon), which were unclassified at genus level, but these bins were removed from Figs. 1 and 2 to aid in visualization and to focus on the identified genera. Edge thickness represents the strength of the correlation. Blue edges represent positive correlations, and Orange edges represent negative correlations. Green nodes represent metabolite classes and may contain more than one metabolite feature (hence why there may be multiple edges between two nodes), and Cyan nodes represent bacterial genera. Check Table 6 at the end of this article for the key to the abbreviations used in Figs. 1 and 2.

**Fig. 2:**
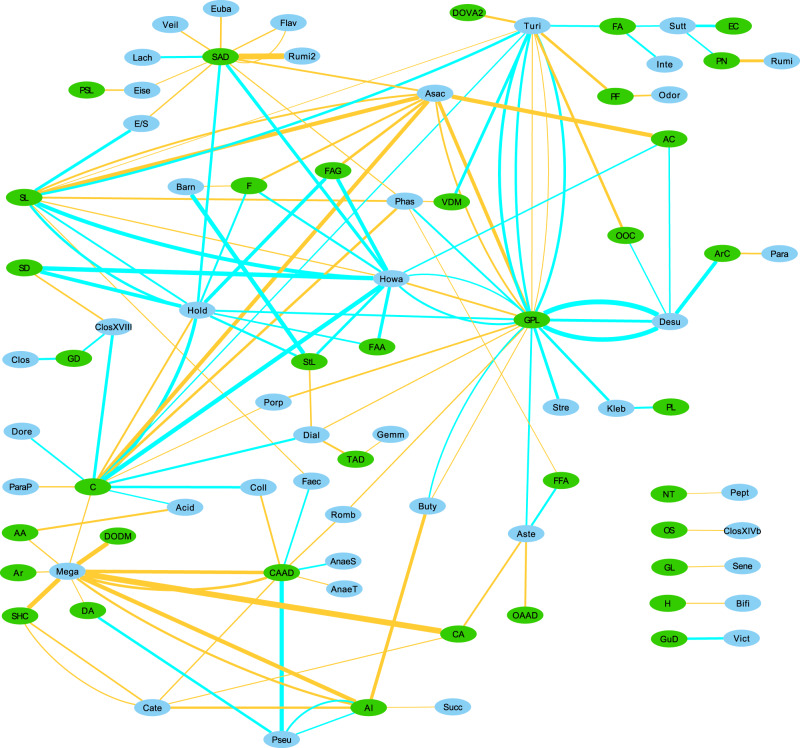
Network of within-Controls’ correlations. Network of within-Controls’ correlations between bacterial genera and metabolite classes. Supplementary Tables 1 and 2 contain correlations with bins composed of more than one genus (or higher taxon) which were unclassified at genus level, but these bins were removed from Figs. 1 and 2 to aid in visualization and to focus on the identified genera. Edge thickness represents the strength of the correlation. Blue edges represent positive correlations, and Orange edges represent negative correlations. Green nodes represent metabolite classes and may contain more than one metabolite feature (hence why there may be multiple edges between two nodes), and Cyan nodes represent bacterial genera. Check Table 6 at the end of this article for the key to the abbreviations used in Figs. 1 and 2.

The within-PD analysis at genus level identified a total of 176 correlation pairs, while within-Controls analysis produced 202 pairs (Supplementary Tables 1 and 2, respectively). As can be seen, there is some overlap in the taxa and metabolites represented in the two groups, but overall there are substantial differences in the bacterial taxon-metabolite pair associations. To aid in the identification of possible links between metabolite classes and bacteria, we produced network figures for both within-PD and within-Controls results at genus level, using metabolite classes. (Figs. 1 and 2). At family level, the within-PD analysis identified a total of 67 correlation pairs, while the within-Controls analysis identified a total of 85 pairs (Supplementary Tables 3 and 4, respectively). Finally, the within-PD analysis at phylum level identified a total of 17 correlation pairs and the within-Controls analysis identified a total of 6 pairs (Supplementary Tables 5 and 6, respectively).

**Fig. 3:**
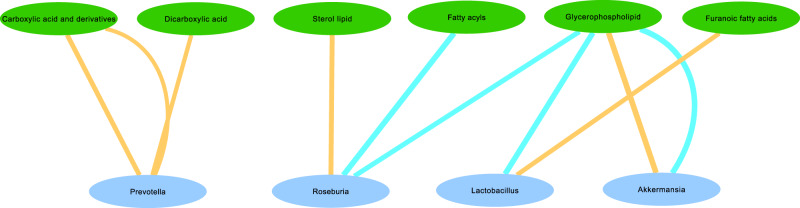
Network of selected within-PD correlations. Network of within-PD correlations between bacterial genera and metabolite classes using selected results (Table 4). Edge thickness represents the strength of the correlation. Blue edges represent positive correlations, and Orange edges represent negative correlations. Green nodes represent metabolite classes, and Cyan nodes represent bacterial taxa.

To further focus the study, we trimmed the correlation pairs down to only those containing bacterial taxa that were (i) not unclassified at the target taxonomic level, (ii) differentially abundant between PD and Control groups at one or both of the two time points of sample collection in a previous study using the same subject data^14^, and (iii) taxa that were systematically reported previously in the PD microbiome literature as being differentially abundant between PD and Control groups^3,14^ (Table 4). The bacterial abundance data used in the present article corresponds to the second time point of sample collection in Aho et al.^14^. To aid in visualizing the relationships, a third within-PD network figure was produced, using genus-level data and metabolite classes as before, but limited to the trimmed correlation pairs (Fig. 3).

**Table 4:** Selected results.

	Metabolite Peak ID	Metabolite MSI 3 ID	Class	p2.5	post. mean corr.	p97.5	Taxon Diff Abund Direction Consensus
							(Aho et al. 2019/Boertien et al. 2020)
Bacterial Genus:							
*Within-PD analysis:*							
Prevotella	X7253	Proline	Carboxylic acid and derivatives	−0.5146	−0.3307	−0.1186	Decreased in PD/same
Prevotella	X7287	Tartronic acid	Dicarboxylic acid	−0.5025	−0.3148	−0.1066	Decreased in PD/same
Prevotella	X7206	N-formyl-methionine	Carboxylic acid and derivatives	−0.4957	−0.3063	−0.0929	Decreased in PD/same
Roseburia	X499	3-Deoxyvitamin D3	Sterol lipid	−0.5249	−0.3348	−0.1159	Decreased in PD/same
Roseburia	X423	PC(P-20:0/18:2(9Z,12Z))	Glycerophospholipid	0.1034	0.3070	0.4970	Decreased in PD/same
Roseburia	X370	3-octadecylenic acid	Fatty acyls	0.1231	0.3300	0.5208	Decreased in PD/same
Lactobacillus	X6092	3,4-dimethyl-5-carboxyethyl-2-furanpentanoic acid	Furanoic fatty acids	−0.5270	−0.3246	−0.0991	Increased in PD/mostly increased in PD
Lactobacillus	X7038	PE(20:2(11Z,14Z)/22:5(4Z,7Z,10Z,13Z,16Z))	Glycerophospholipid	0.0916	0.3260	0.5291	Increased in PD/mostly increased in PD
Akkermansia	X7038	PE(20:2(11Z,14Z)/22:5(4Z,7Z,10Z,13Z,16Z))	Glycerophospholipid	−0.5454	−0.3572	−0.1350	NA/increased in PD
Akkermansia	X6564	PS(19:0/0:0)	Glycerophospholipid	0.1056	0.3305	0.5304	NA/increased in PD
*Within-Controls analysis:*							
Bifidobacterium	X7163	2-amino-2-deoxy-glucose	Hexoses	−0.5043	−0.3109	−0.0854	Increased in PD/mostly increased in PD
Bacterial Family:							
*Within-PD analysis: *							
Bifidobacteriaceae	X7145	PI-Cer(t20:0/22:0(2OH))	Glycerophospholipid	0.0948	0.3069	0.4977	Increased in PD/mostly increased in PD
Pasteurellaceae	X6860	PE(16:0/P-18:1(11Z))	Glycerophospholipid	0.1106	0.3521	0.5620	Decreased in PD/NA
Lactobacillaceae	X32	(−)-Jolkinol B	Chemical	−0.5073	−0.3027	−0.0664	Increased in PD/mostly increased in PD
Lactobacillaceae	X7038	PE(20:2(11Z,14Z)/22:5(4Z,7Z,10Z,13Z,16Z))	Glycerophospholipid	0.0823	0.3074	0.5096	Increased in PD/mostly increased in PD
Verrucomicrobiaceae	X7038	PE(20:2(11Z,14Z)/22:5(4Z,7Z,10Z,13Z,16Z))	Glycerophospholipid	−0.5430	−0.3579	−0.1496	NA/increased in PD
Verrucomicrobiaceae	X6564	PS(19:0/0:0)	Glycerophospholipid	0.1288	0.3413	0.5297	NA/increased in PD
Erysipelotrichaceae	X7253	Proline	Carboxylic acid or derivative	0.1616	0.3715	0.5648	NA/mostly increased in PD
*Within-Controls analysis:*							
Bifidobacteriaceae	X7163	2-amino-2-deoxy-glucose	Hexoses	−0.5127	−0.3171	−0.1049	Increased in PD/mostly increased in PD
Lactobacillaceae	X7183	Beta-alanine	Carboxylic acid	−0.5079	−0.3062	−0.0822	Increased in PD/mostly increased in PD
Lactobacillaceae	X7081	PS(19:0/22:6(4Z,7Z,10Z,13Z,16Z,19Z))	Glycerophospholipid	0.1223	0.3725	0.5795	Increased in PD/mostly increased in PD
Enterobacteriaceae	X485	SM(d18:1/21:0)	Sphingolipid	0.1140	0.3280	0.5281	NA/mostly increased in PD
Enterobacteriaceae	X430	PC(P-20:0/18:3(6Z,9Z,12Z))	Glycerophospholipid	0.1292	0.3299	0.5325	NA/mostly increased in PD
Bacterial Phylum:							
*Within-PD analysis:*							
Verrucomicrobia	X7038	PE(20:2(11Z,14Z)/22:5(4Z,7Z,10Z,13Z,16Z))	Glycerophospolipid	−0.5306	−0.3414	−0.1231	NA/increased in PD
Verrucomicrobia	X6564	PS(19:0/0:0)	Glycerophospolipid	0.1064	0.3345	0.5302	NA/increased in PD

Selected results based on taxa previously reported in the literature at genus, family, and phylum levels. The last column presents a consensus on direction of effect based on previous reports: before the slash (/), we report the result obtained in Aho et al.^14^ using the same bacterial data as in the present study; after the slash, we report the consensus reported by Boertien et al.^3^ (see that study for details). NA means that no result for that taxon is available in that study^14^.

In the Helsinki cohort, six bacterial genera were previously reported as being differentially abundant (selected for the present article at an alpha threshold of statistical significance of 0.05 from the original 0.1), using one or more statistical methods^14^, between Control and PD groups at one or both time points, namely: *Bifidobacterium*, *Roseburia*, *Prevotella*, *Blautia*, *Lactobacillus*, and *Clostridium XIVa*. All these genera, except for *Clostridium XIVa*, have also been reported as being differentially abundant in previous publications contrasting a control group to PD patients^14^. Of these, *Prevotella*, *Bifidobacterium*, *Roseburia*, and *Lactobacillus* are also found to be correlated with one or more metabolite features in our genus-level analysis (Table 4).

Other differentially abundant bacterial genera reported previously in the PD microbiome literature^3^ besides those referred to in Aho et al.^14^ have also been found covarying with metabolite features in our dataset, and we also used that information for the purpose of focusing our study’s results. At genus level, *Akkermansia*, *Bifidobacterium*, *Faecalibacterium*, *Prevotella*, *Lactobacillus*, and *Roseburia* were reported multiple times in the literature, with only *Akkermansia* and *Faecalibacterium* not being reported in the Aho et al.^14^ study as being differentially abundant (Table 4).

In the Aho et al.^14^ study, seven bacterial families were reported as being differentially abundant between PD and Control groups at one or both time points, namely: *Bifidobacteriaceae, Prevotellaceae, Rikenellaceae, Lachnospiraceae, Pasteurellaceae, Lactobacillaceae*, and *Puniceicoccaceae*. All these families, except for *Puniceicoccaceae*, have also been reported as being differentially abundant in previous publications contrasting a control group to PD patients^14^. Three of them showed correlations with one or more metabolite features in our family-level analysis (Table 4). Boertien et al.^3^ also reported on the bacterial families most commonly found to be differentially abundant, namely *Bifidobacteriaceae*, *Prevotellaceae*, *Lachnospiraceae*, *Lactobacillaceae, Verrucomicrobiaceae, Enterobacteriaceae, Erysipelotrichaceae*, and *Ruminococcaceae*, with the last four families not being found to be differentially abundant at any time point in our cohort^14^ (Table 4).

For this study, we have also analysed our data at phylum level, unlike in Aho et al.^14^, and found correlations between various metabolites and the phyla *Lentisphaerae, Verrucomicrobia, Synergistetes*, and *Tenericutes* (Supplementary Tables 5 and 6). Some phyla recurrently found to be differentially abundant between PD and Control groups are *Verrucomicrobia, Firmicutes*, and *Bacteroidetes*^3^, although of those reported only *Verrucomicrobia* yielded correlations with metabolite features in our analysis (Table 4).

## Discussion

Using mummichog approach, we have shown in this study that the identified serum metabolome differences in PD have functional significance on over 20 pathways, including carnitine shuttle, vitamin E metabolism, glycerophospholipids, sphingolipids, fatty acids, and aminoacyl-tRNA biosynthesis. In a separate study, we have demonstrated that carnitine shuttle, sphingolipids, and fatty acids pathways change in Parkinson’s sebum—these are within the 20 pathways enlisted above detected in serum in this study^19^.

The changes observed in pathways associated with sphingolipid metabolism may indicate a key shift in cell signalling and regulation. Dysregulation of sphingolipids is known to be associated with α-synucleinopathy^20,21^, changes in lysosomal metabolism, and in mitochondrial metabolism observed in PD^22,23^. Sphingolipids have shown strong associations with many neurodegenerative conditions as recently reviewed by Alessenko and Albi^24^. In addition to altered sphingolipid metabolism in plasma, metabolomics and lipidomics studies in PD have shown changes in ceramides, sphingosine and sphingosine-1-phosphate in particular^25,26^. It is not surprising, given that one of the top pathways where notable changes are seen in this study is linked to glycerophospholipid metabolism. Glycerophospholipids and sphingolipids are well-known as the ‘ying and yang’ of lipotoxicity in metabolic disease^27^. The dysregulation of these complementary and opposed forces in the metabolome leads to lipotoxicity seen in many metabolic diseases. It can thus be speculated that such lipotoxic insult may be one of the underlying pathophysiologies of PD, as measured in serum.

Decreased long-chain acylcarnitines due to insufficient β-oxidation has been shown to carry potential for early diagnosis of Parkinson’s^28^, especially 12–14 long chain acylcarnitines. In recent work studying the gut microbiome, Rosario et al.^29^ have shown the role of bacterial folate and homocysteine metabolism in PD. Higher numbers of bacterial mucin and host degradation enzymes were linked to the manifestation of PD. The contribution of bacterial folate metabolism to human metabolic regulation is not entirely clear. Folate is an essential vitamin B, that maintains methylation reactions. The liver, via many methylation reactions in post-translational modifications, regulates the synthesis of hormones, creatine, carnitine, and phosphatidylcholine^30^. If methylation capacity is compromised due to an alteration in folate metabolism, there may be impaired phosphatidylcholine synthesis along with shunted or disrupted carnitine shuttle observed in our results. Altered carnitine metabolism, fatty acids, and steroid metabolism were also observed in a metabolomics profiling study recently reported^31^. Several studies have reported decreased levels of carnitine and acylcarnitines in plasma from PD patients^28,32,33^; however, according to Jiménez-Jiménez et al.^34^ no changes were observed in acylcarnitine levels in plasma or cerebrospinal fluids of PD participants. Thus, there is no clear evidence of direction in which carnitines are expressed but there is much research evidence that suggests a link between perturbations in carnitine shuttle owing to protective mechanism of acylcarnitines leading to changes in other fatty acids and eventually the lipid make-up in PD. Researchers have shown molecules such as LDL and HDL to have direct association with sebum and related diseases such as acne^35^ and demonstrated that lipid metabolism is not just a diet processing effect, but a complex interaction that affects lipid uptake from the gut, biosynthesis in liver and sequestration in tissues including on skin^36^. These results in serum metabolome could be indicative of link between gut microbiome, serum metabolome and sebum metabolome.

Energy metabolism is highly regulated by facilitation of long chain fatty acid β-oxidation. Also, in serum from frail^11^ elderly participants without Parkinson’s, dysregulation of carnitine shuttle and vitamin E metabolism was observed when compared to similarly aged resilient individuals^37^. Thus, perturbation of carnitine shuttle and vitamin E, along with fatty acids in serum metabolome may indicate a significant change in energy metabolism during PD. Further, Vitamin E’s role as a protective factor against Parkinson’s has been extensively studied along with vitamin C for therapy of early onset PD^38–41^. Serum metabolome based on pathway analysis presented in this study indicates changes broadly in energy metabolism and lipid metabolism in PD. These disruptions have been recently reported in other biofluids such as sebum^19^ and CSF^42^ and the community is increasingly recognising the role of lipid dysregulation, well summarized in these reviews^20,43,44^. We used knowledge from our microbiome analysis to investigate if these metabolic shifts were entirely endogenous or were also partly contributed to by changing gut microbiome in Parkinson’s disease.

Regarding the correlations between bacterial taxa and metabolite features, and given that metabolite MSI 3 ID is a putative identification, we will mostly focus the following discussion at the level of metabolite classes. When mentioning specific bacterial taxa in terms of correlation results, we will report between brackets if the taxon is always (or usually) reported in the literature as being over- or underrepresented in PD (see the last column in the Table 4 for details), as well as the signal of the correlation detected in the present study.

At all bacterial taxonomic levels investigated in this study, the most commonly detected correlations were found with putative metabolites in the glycerophospholipid class and with lipids in general. This is the case both in our non-trimmed results (Supplementary Tables 1–6) and in the trimmed results focusing only on bacterial taxa found in the previous literature as being differentially abundant between Controls and PD cases (Table 4). Specifically, in the within-PD analysis and focusing the discussion on the trimmed results, we find correlations between various glycerophospholipids and *Roseburia* (decreased in PD; positive correlation)*, Lactobacillus* (increased in PD; positive correlation)*, Akkermansia* (increased in PD; one positive and one negative correlation)*, Bifidobacteriaceae* (increased in PD; positive correlation), *Pasteurellaceae* (decreased in PD; positive correlation), *Lactobacillaceae* (increased in PD; positive correlation), *Verrucomicrobiaceae* (increased in PD; one positive and one negative correlation), and *Verrucomicrobia* (increased in PD; one positive and one negative correlation) (Table 4). *Akkermansia*, *Verrucomicrobiaceae*, and *Verrucomicrobia* share the same positive and negative correlations with two metabolite features. *Lactobacillus* and *Lactobacillaceae* correlate positively with the same metabolite feature.

With the exception of *Roseburia* and *Pasteurellaceae*, all these taxa are usually found to be overrepresented in PD, and are mostly positively correlated with various glycerophospholipids, which are also found in our analysis to be mostly overrepresented in PD (Table 2). This is not the case in the within-controls analysis, in which the only detected correlations with glycerophospholipids are with *Lactobacillaceae* (positive correlation with a different metabolite) and *Enterobacteriaceae* (also a positive correlation with a different metabolite) (Table 4). The genus-level network figures for PD and Controls (Figs. 1 and 2) also indicate the existence of possible alterations in bacterial metabolic dynamics in PD. Overall, these results suggest that these bacterial taxa, which have been found to be overabundant in PD in several studies, may be associated for the most part with an increase in glycerophospholipid abundance in PD.

All of these glycerophospholipids have an endogenous (human host) origin and are linked to cell signalling, lipid peroxidation, and lipid metabolism. These phospholipids are the main component of cell membranes in all known living systems, and play roles in various biological processes, including signal induction and acting as transporters. Interestingly, there are several genetic factors directly or indirectly related to glycerophospholipid metabolism, such as *PLA2G6*^20^, that are associated with PD risk (*PLA2G6* is the cause of early-onset PARK14-linked dystonia- parkinsonism^45,46^). Alpha-synuclein, characteristically found in aggregates within Lewy bodies in the brains of PD patients, directly binds to negatively charged phospholipids in the cells’ lipid membranes, and exhibits preferential binding to small lipid vesicles^47^. The binding of alpha-synuclein to lipid membranes can also lead to alterations in their bilayer structure that can induce the formation of those lipid vesicles^48^. Very importantly in the PD context, these interactions between lipid membranes and alpha-synuclein affect its rate of aggregation, and can lead to disruption of membrane integrity both in vitro and in vivo^49^. It has also been shown that the association of soluble alpha-synuclein with planar lipid bilayers results in the formation of aggregates and small fibrils^50^. Exposure to docosahexaenoic acid (DHA), which accounts for 60% of glycerophospholipid esterified fatty acids in the plasma membrane, gradually assembles alpha-synuclein into amyloid-like fibrils, with the notable feature that DHA itself becomes part of the aggregate^51^. Notably, alpha-synuclein gene expression is increased with elevated DHA intake, and the resulting oligomers are toxic to cells^52,53^. Alpha-synuclein also binds with specific phospholipids in mitochondrial membranes, modulating the efficiency of mitochondrial energy production^54^, with various mitochondrial phospholipids appearing to have an effect on alpha-synuclein toxicity^20^. Thus, the interaction between alpha-synuclein and various phospholipids and their metabolism may play an important role in PD pathogenesis, and gut microbiota may be implicated in these interactions.

We also detected correlations with other lipids in the within-PD analysis. *Roseburia* (decreased in PD) negatively correlates with a sterol lipid, probably of dietary origin. *Roseburia* is also positively correlated with a metabolite in the fatty acyl class, possibly also associated with diet. *Lactobacillus* (increased in PD) is negatively correlated with a furanoic fatty acid, which is associated with cell signalling, lipid peroxidation, lipid metabolism, and lipid transport metabolism, with dietary, human, and/or bacterial origin. Half of the metabolites from the fatty acyl class were found to be overrepresented in PD in the selected 139 metabolite features in our blood serum data (Table 2), but overall underrepresented in the PD sebum data from Sinclair et al.^19^.

In the within-controls analysis we detected a positive correlation between *Enterobacteriaceae* (increased in PD) and a sphingolipid, which is associated with membrane stabilization, lipid peroxidation, and lipid metabolism, of endogenous origin. In our study, this metabolite feature is slightly decreased in PD (Table 2) and no correlation is found between it and PD-linked bacterial taxa in the within-PD analysis.

Although the interpretation of these results regarding lipids in general is not suggestive of a particular pattern as in the case of glycerophospholipids, it is nevertheless interesting that virtually all correlations, positive or negative, with lipids are detected in the PD group, with most lipids detected in our study being overrepresented in PD relative to the control group (Table 2). Also interesting is that the majority of the identified correlations between metabolite features and the trimmed bacterial taxa list are with lipids, with relatively few other metabolite groups represented in the results (Table 4). The importance of this link between lipid metabolism and PD can’t be overstated: as mentioned earlier in the context of phospholipids, recent research shows that alpha-synuclein binds preferentially to specific lipid families and molecules, and that the latter promote alpha-synuclein interaction with synaptic membranes and affect alpha-synuclein oligomerization and aggregation. These same lipid-protein complexes also affect lipid metabolism by interfering with the catalytic activity of lipid enzymes in the cytoplasm and lipases in lysosomes. Lipid compositional alterations in PD have also been reported in brain and plasma, as well as linked to oxidative stress, inflammation, and progressive neurodegeneration through pro-inflammatory lipid mediators (see Alecu et al.^20^ for a full review on the role of lipids in PD). The link between lipids and bacteria in PD, if any, would probably consist of the bacterial modulation of lipid intake through diet and its differential effect on the bioavailability of those lipids in the host. The results of our study, by establishing associations between bacterial taxa found to be differentially abundant between Controls and the PD group and lipid metabolites present in serum that are themselves predictive of PD, suggests that such a scenario could have a role in PD pathology and development.

Further correlations with putative metabolites in other classes have also been detected in our study, in particular in the hexoses class and carboxylic acid or derivatives class. In the hexoses case, two negative correlations for the same metabolite feature were found for *Bifidobacterium* and *Bifidobacteriaceae* (both taxon levels increased in PD; Table 4). These two correlations are only found in the within-controls analysis. The metabolite is probably endogenous in origin and is involved in sugar metabolism shunts, diverting a proportion of glucose from the main glycolytic path and returning metabolites at the level of triose phosphate and fructose 6-phosphate.

Finally, a positive correlation in the within-PD analysis is found between a metabolite feature in the carboxylic acid or derivatives class and the bacterial family *Erysipelotrichaceae*, which is mostly found in previous studies to be overrepresented in PD. This metabolite feature is tentatively identified as proline and may have a microbiome or endogenous source. Four different metabolite features found to be predictive of PD in our data are tentatively identified as proline (Table 2), and in all cases show a slight decrease in mean abundance in PD. Interestingly, L-proline can act as a weak agonist for glycine and glutamate receptors^55^, like NMDA, AMPA, and kainite. Both glutamate and glycine are neurotransmitters. Although that is not the case in our data, proline has been reported previously as being overrepresented in PD^31^ and is known to be linked to protein metabolism and structure, cell differentiation, conceptus growth and development^56^, and gut microbiota community re-equilibration in cases of dysbiosis, with L-proline dietary supplementation being known to affect gut microbial composition and gut concentrations of several bacterial metabolites^57^. One of the detected correlations with *Prevotella* also involves (putative) proline. *Prevotella* and *Prevotellaceae*, when detected in PD studies, are usually found decreased in PD. In this study it correlates negatively with proline, which is found to be slightly underrepresented in the PD group.

In conclusion, we designed our study to investigate, as broadly as possible, the existence of associations between bacteria and metabolite abundances in PD. To this end, we used a data-driven, hypothesis-generating approach based on untargeted metabolomics data. It is our hope that the results can a posteriori be used to build more focused studies of either an observational or experimental nature to explore our putative findings. Circa 7500 serum metabolite features were detected by gas chromatography and liquid chromatography using untargeted metabolomics. Of these, 139 were deemed to be particularly predictive for PD status. For the most part they are related to lipid metabolism, and are also mostly overrepresented in PD. The evidence for metabolic differences in PD is related to carnitine shuttle, vitamin E metabolism, glycerophospholipids, sphingolipids, and fatty acids, suggesting alterations in PD related to energy and lipid metabolism. Our results indicate that the abundance of several gut bacterial genera (e.g. *Prevotella, Bifidobacteria, Akkermansia, Lactobacillus, Roseburia*) correlates with the abundance of several of these metabolite features, and thus may be implicated in these metabolic alterations in PD.

One limitation of this study, like in many other metabolomics clinical studies, could be the variation introduced by drug usage. We have analysed possible associations between the metabolite features against several medications (e.g. LEDD score) and found no clear indication of drug effects. However, without a targeted metabolomics study, it is hard to rule out such effects. We may have these drugs or their breakdown products in our data, but we cannot target them specifically due to the unknown masses of all possibly relevant drug metabolites. We hope that untargeted metabolomics studies like the present one could, with their broad hypothesis-generation approach, serve as a basis for future targeted metabolomics studies that could better deal with potential sources of variation such as drugs or their metabolic products.

## Methods

### Study subjects, clinical data, and sampling

This study was conducted in accordance with the Declaration of Helsinki and was approved by the ethics committee of the Hospital District of Helsinki and Uusimaa. All participants provided written informed consent.

The present study uses bacterial abundance data that was used in a previously published study by Aho et al.^14^. The study subjects and associated clinical data in the present study is similar to the data referred to previously in that study as “follow-up” timepoint, with minor changes specific to the present study: of the original 128 subjects, 61 control subjects and 63 PD patients were used in the present study, i.e. 3 control subjects and 1 PD subject less than in the original cohort (C75, C82, C123, and P119). This difference in sample numbers was due to insufficient metabolite data available to perform the study.

For DNA sequencing, the stool samples were collected at home by the study subjects using tubes containing DNA stabilizer (PSP Spin Stool DNA Plus Kit, STRATEC Molecular), which were stored for a maximum of three days in a freezer until transport. At the clinic, the received samples were stored at −80 °C and later transferred to the sequencing centre, where they were also stored at −80 °C until further processing^14^.

For serum samples, blood was drawn at the study visit and, after processing, immediately transferred to −20 °C and subsequently to −80 °C. Samples were shipped overnight on dry ice from Helsinki to Manchester for analysis.

### Sample preparation and metabolomics methods

#### Metabolomics sample preparation

Untargeted metabolite profiling was performed on serum samples that were collected from participants and stored at −80 °C prior to analysis. Complementary coverage of metabolites was obtained using ultra-high performance liquid chromatography mass spectrometry (UHPLC-MS) and gas chromatography mass spectrometry (GC-MS). The procedures were adapted from the Dunn^58^ and Begley^59^ protocols as summarized here:

Metabolites were extracted from the serum samples by individually adding 400 µL of cold methanol to 200 µL of serum. This was followed by vortexing and centrifugation (17,500 × *g*) to yield a metabolite rich supernatant that was split into two aliquots and lyophilised for 12 h. Resultant metabolite pellet was stored at −80 °C until analysis. A pooled QC standard was also generated by combining 20 µL aliquots of each sample into a pooled vial with subsequent 200 µL aliquots from the pool, being extracted identical to each sample.

#### LC-MS method parameters

Processed metabolite pellets were defrosted at 4 °C and subsequently reconstituted in 100 µL of 95:5 H_2_O:MeOH (*v/v)*. UHPLC-MS analysis was performed using an Accela UHPLC with cooled auto sampler system coupled to an electrospray LTQ-Orbitrap XL hybrid mass spectrometer (ThermoFisher, Bremen, Germany). Analysis was carried out in positive and negative ESI modes while samples were completely randomised to negate for any bias. The mobile phases and gradient elution profile were as tabulated in Table 5. From each sample vial, 10 µL of the extract was injected onto a Hypersil GOLD UHPLC C18 column (length 100 mm, diameter 2.1 mm, particle size 1.9 µm, Thermo-Fisher Ltd. Hemel Hempsted, UK) held at a constant temperature of 50 °C with a solvent flow rate of 400 µL min^−1^.

**Table 5:** Mobile phases and gradient elution profile.

Time (min)	Mobile Phase A (95:5 H_2_O:MeOH with 0.1% Formic acid) composition	Mobile Phase B (95:5 MeOH:H_2_O with 0.1% Formic acid) composition
0	100	0
1	95	5
12	5	95
20	5	95
22	95	5
25	95	5

Prior to analysis, LTQ-Orbitrap XL was calibrated according to manufacturer’s instructions using caffeine (20 µg mL^−1^), the tetrapeptide MRFA (1 µg ml^−1^) and Ultramark 1621 (0.001%) in an aqueous solution of acetonitrile (50%), methanol (25%) and acetic acid (1%). The data acquisition was performed in centroid mode with 30 K mass resolution and scan rate of 400 ms per scan. The masses were measured between 100 and 1200 *m/z* range with source gases set at sheath gas = 40 arb units, aux gas = 0 arb units, sweep gas = 5 arb units. The ESI source voltage was set to 3.5 V, and capillary ion transfer tube temperature set at 275 °C.

#### LC-MS data processing

Xcaliber software (v.3.0; Thermo-Fisher Ltd. Hemel Hempsted, UK) was used as the operating software for the Thermo LTQ-Orbitrap XL mass spectrometer. Data processing was initiated by the conversion of the standard UHPLC*.raw* files into the*.mzML* format using Proteowizard^60^. Subsequently, peak picking was carried out in RStudio^61^ using the XCMS^62^ package for data deconvolution (http://masspec.scripps.edu/xcms/xcms.php). The output data was a matrix of mass spectral features with accurate *m/z* and retention time pairs. Any missing values after deconvolution were replaced using k-nearest neighbours algorithm. Peaks with relative standard deviation of more than 20% within pooled QCs were removed. The remaining data was normalised with total ion count to account for injection to injection signal variations, log_10_ transformed, and pareto scaled prior to statistical analysis.

#### GC-MS method parameters

Analysis of serum samples was also carried out on a Agilent 7250 GC-Time-of-Flight mass spectrometer coupled to a Gerstel-MPS autosampler. Two step derivatization of metabolite pellets thawed at 4 °C was carried out as described in the Begley protocol^59^. The source temperature was set to 230 °C and quad temperature was at 150 °C. The total run time was 25 min for 10 µL sample injected each time. The sample was injected in split mode with 20:1 split ratio and split flow of 20 mL per minute. Agilent CP8944 VF-5 ms column was used for separation (30 m × 250 µm × 0.25 µm). With a 5 min solvent delay at the start of run, gradient elution method was used to elute and separate analytes from serum. The oven temperature was ramped from 70 °C to 300 °C with an increase of 14 °C per minute. At 300 °C the temperature was held for 4 min before dropping back to starting conditions.

#### GC-MS data processing

The raw data files were in Agilent*.D* format that were converted to*.mzML* format using Proteowizard^60^. Peak picking was carried out in RStudio^61^ using an in-house script for the eRah^63^ package for GC-MS peak picking and deconvolution. The peaks were annotated using eRah’s MassBank library. Any missing values after deconvolution were replaced using k-nearest neighbours algorithm. Peaks with relative standard deviation of more than 20% within pooled QCs were removed. The remaining data was normalised with total ion count to account for injection to injection signal variations, log_10_ transformed, and pareto scaled prior to statistical analysis.

All metabolites successfully annotated within both the LC-MS and GC-MS analysis were assessed and scored at *MSI level 3 putative identification* according to rules set out by the Chemical Analysis Working Group of the Metabolite Standards Initiative^18^.

### Sample preparation and DNA sequencing

DNA extraction was performed according to the manufacturer’s instructions. We then amplified the V3-V4 regions of the bacterial 16 S rRNA gene, using two technical replicates (25 μL reactions) per biological sample, and a mixture of the universal bacterial primers 341F1–4 (5′ CCTACGGGNGGCWGCAG 3′) and 785R1–4 (5′ GACTACHVGGGTATCTAATCC 3′) with partial Illumina TruSeq adapter sequences added to the 5′ ends (F1; ATCTACACTCTTTCCCTACACGACGCTCTTCCGATCT, F2; ATCTACACTCTTTCCCTACACGACGC TCTTCCGATCTgt, F3; ATCTACACTCTTTCCCTACACGACGCTCTTCCGA TCTagag, F4; ATCTACACTCTTTCCCTACACGACGCTCTTCCGATCTtagtgt and R1; GTGACTGGAGTTCAGACGTGTGCTCTTCCGATCT, R2; GTGACT GGAGTTCAGACGTGTGCTCTTCCGATCTa, R3; GTGACTGGAGTTCAGACGT GTGCTCTTCCGATCTtct, R4; GTGACTGGAGTTCAGACGTGTGCTCTTCCGA TCTctgagtg)^14^. The small letters in the above sequences are additional nucleotides introduced for purposes of mixing in the sequencing process. We performed two-step PCRs followed by quantification, pooling, and purification^14^. The resulting PCR products were then sequenced with Illumina MiSeq (v3 600 cycle kit), with 325 bases and 285 bases for the forward and reverse reads, respectively.

### Bioinformatics and statistical data analysis

#### Statistical analysis of metabolomics data

In this untargeted profiling study, we detected a total of 7585 features combined between LC-MS positive ionisation mode, LC-MS negative ionisation mode and GC-MS data. After processing the raw data and applying QC based relative standard deviation filtering, in LC-MS positive ionisation mode 5897 features remained, and in LC-MS negative ionisation mode data 1260 features remained. In GC-MS data 428 features were retained for statistical analysis. As described earlier in this article, these features were scaled and transformed prior to any statistical analysis.

##### (i) Classification by disease status

Individual dataset from LC-MS positive mode, LC-MS negative mode, and GC-MS were first analysed separately and then standardised combined data was used to generate a model based on the whole metabolome measured by 7585 features in total. Individual datasets from each technique were analysed using partial least squares discriminant analysis (PLS-DA) to classify between PD and control using the Metaboanalyst package in R. The models were validated by 60:40 split of data with 250 bootstraps and also by comparing correct classification rates with permuted models (Supplementary Table 7 (MODELS)).

In order to investigate the classification of samples into PD and control, support vector machines (SVM) were used on 7585 metabolite features. Using Python via Orange user interface, SVM models were generated for this analysis. The data were pre-treated as described earlier in this article. The data were then split into train (60% data) and test (40% data), and resampling was repeated 100 times. The SVM model was generated with RBF-kernel, cost (C) was set to 1.5, and regression loss epsilon was set at 0.10. We accounted for potential effects of hypercholesterolaemia, gender, age at sampling, BMI, defecations per week, weekly stool characteristics average, clinical scores (GDS15, MMSE, NMSQ, NMSS, RBDSQ, Rome-III constipation score, SDQ, Wexner total, Progression score, and Rome-III IBS criteria) as well as 68 dietary and lifestyle related variables as described in Supplementary table 7 (MODELS). The top 10% variables were selected after ranking them by ReliefF algorithm, and models were regenerated to rule out the possibility of an effect due to a large number of metabolite features.

##### (ii) Key predictive metabolite feature selection

To select metabolite features (i.e., features predictive of PD status) contributing to the SVM models, the mSVM-RFE algorithm was used^64^. The iterative algorithm worked backwards from an initial set of features consisting of all the metabolite features in the dataset. At each iterative round, firstly a simple linear SVM was fitted, then features were ranked based on their weights in the SVM solution and lastly, the algorithm eliminated the feature with the lowest weight. In order to stabilize these feature rankings, at each iteration cross validation resampling was used. By using k-fold cross validation (k = 10) multiple SVM-RFE iterations were carried out. From the resultant ranked feature list, the top 10% of the features were selected (the 139 key predictive metabolite features) for further interpretation as they contributed the most towards SVM models.

##### (iii) Effects of PD medications, UPDRS-III score, and time since onset of motor symptoms on key metabolite features selected

During the selection of the key predictive metabolite features, potential effects from clinical variables like PD medication could not be taken directly into account in those models, since they are restricted to the PD group. However, such an approach would leave open the question regarding possible effects of those variables on the metabolite predictors. Thus, to investigate the potential effects of PD medications, UPDRS-III score, and time since motor symptoms on PD vs HC classification (i.e. the 139 metabolite features), and to investigate associations of drug dosages to the key metabolite features, partial least squares and logistic regression were carried out within-PD for continuous and categorical responses, respectively. The variables that had continuous scaled values were subjected to partial least squares regression with 20 PCs, for 1000 iterations. The variables that had categorical values were subjected to logistic regression with ridge penalization with cost value of 1. All partial least squares and logistic regression models were validated by leave-one-out cross-validation (LOOCV). Partial least squares R^2^ and root mean squared error (RMSE) and logistic regression classification accuracy were used for model evaluation (Supplementary Table 7 (MODELS)). The top 10% of variables were selected after ranking them by ReliefF algorithm, and models were regenerated to rule out the possibility of an effect due to a large number of metabolite features. Our results indicate that the choice of 139 metabolite features was not affected by PD medication use or dosage. One possible exception could be COMT inhibitors, which may have a weak effect and may be associated with the PD metabolome in serum: using the 139 key metabolites, we were able to classify between a PD subject with and without COMT inhibitor treatment with an accuracy of 63%, which is low. Thus, these results suggest by extension that drug use did not affect the choice of the 139 key predictive metabolite features in any substantial way during the HC vs PD predictive feature analysis. Supplementary Table 7 (MODELS) includes information about variables and adjusted variables, for each model.

##### (iv) Effects of clinical variables on metabolome within PD

We investigated potential effects and association of clinical variables within the PD cohort. All metabolite features (not just the key predictive metabolite features) were regressed against clinical features of Parkinson’s viz. GDS-15^65^ (depression), MMSE^66^ (cognition), NMSS^67^ (non-motor symptoms), RBDSQ^68^ (REM-sleep behaviour disorder), Rome-III constipation score^69^, Rome-III IBS status, Wexner score^70^ (constipation), SCS-PD^71^ (drooling), SDQ^72^ (dysphagia), UPDRS II-III, Hoeh & Yahr scale, and progression category from Aho et al.^14^, while adjusting for model covariates (see Supplementary Table 7 (MODELS)). All RBDSQ and Progression categories were adjusted for age at sampling and time since motor onset. SCS-PD, SDQ, UPDRS-II, UPDRS-III and Hoehn & Yahr were also adjusted for LEDD. UPDRS-III was additionally adjusted for beta blockers. Wexner score and Rome-III IBS status and Rome-III constipation scores were also adjusted for anticholinergic medication, constipation medication, opioids, and tricyclic medications, as well as dietary fibre intake. SCS-PD was additionally adjusted for anticholinergic and tricyclics. GDS-15 was additionally adjusted for SSRI medications. MMSE was additionally adjusted for anticholinergic and tricyclic medications.

Partial least squares and logistic regression was carried out for continuous and categorical responses, respectively. The features that had continuous scaled values were subjected to partial least squares regression with 20 PCs, for 1000 iterations. The features that had categorical values were subjected to logistic regression with ridge penalization with cost value of 1. All partial least squares and logistic regression models were validated by splitting data into 60:40 training: test sets and resampling for 100 times. Partial least square R^2^ and root mean squared error (RMSE) and logistic regression classification accuracy were used for model evaluation (Supplementary Table 7 (MODELS)). Top 10% of variables (759 variables) were selected after ranking them by ReliefF algorithm, and models were regenerated to rule out the possibility of an effect due to a large number of metabolite features.

##### (v) Pathway analysis

In this data driven approach, we have interrogated data generated from an untargeted profiling study. It is often impractical to identify each peak in a metabolomics profiling study as it could contain upwards to 5000 features in a single sample. To identify them accurately, the only option is to purchase commercial standards and perform MS/MS analysis in both samples and standards and then match fragmentation spectra. This could be relevant when performing a targeted analysis with a defined set of metabolites. Computationally predicted *m/z* based identification alone is not adequate for pathway analysis due to multiple metabolite matches to single *m/z*. Thus, we have employed mummichog analysis that does not depend on identification of metabolites and then mapping on pathways. Instead, mummichog leverages the collective power in the organisation of metabolic networks. If a list of *m/z* values truly reflects a biological activity, the true metabolites that are represented by these *m/z* values should show enrichment on a local structure of a metabolic network. If the measured *m/z* matches to a falsely represented metabolite, the distribution will be observed randomly. The overall significance of mapping and pathway enrichment is estimated by ranking the *p*-values from the real data among the *p*-values from permutation data to adjust for type I error, along with penalisation. Thus, a robust functional metabolic network gives us insight into our data more than identifying a handful of features. Mummichog^73^ (v.1.0.9) pathway analysis was used to predict network activity from pre-processed UHPLC-MS metabolomics data. The full metabolite data set consisting of 5897 and 1260 features from LC-MS positive and negative mode, respectively, was used as an input. Pathway enrichment analysis was performed on annotated 428 GC-MS features using MetaboAnalystR^74^.

#### Data pre-processing for 16S rRNA gene sequence data

The raw sequence data amounted to a total of 34,701,899 reads. In brief, primers were removed from the reads using cutadapt before further processing^14^. We then used mothur to pair, quality trim, taxonomically classify, and finally cluster the reads into OTUs, following mothur’s Standard Operating Procedure (SOP) for MiSeq. The following customizations were made to the SOP parameters: insert = 40 and deltaq = 10 in make.contigs; maxlength = 450 in the first screen.seqs step; start = 6428 and end = 23,440 in the second screen.seqs step; diffs = 4 in pre.cluster. Singleton sequences were also removed with split.abunds (cutoff = 1) before running classify.seqs. The reference databases used were the full-length SILVA alignment release 128 for align.seqs and the RDP 16S rRNA reference (PDS) version 16 for classify.seqs. The final, processed data set (without sequencing blanks) consisted of 18,867 278 reads^14^.

#### Metabolome-microbiome correlation analysis

For correlation analysis between metabolites and bacterial taxa at genus, family, and phylum levels, we used the *fido*^75^ package (v.0.1.13; the package was formerly known as *stray*) for the R Statistical Programming Software^76^ (v.3.6.0). *fido* provides a framework for inferring multinomial logistic-normal models which can account for zeros and compositional constraints, as well as sampling and technical variation present in sequence count data^75^. For the present study we used the function *orthus* from *fido* which enables joint modelling of multivariate count data (e.g. 16S rRNA gene amplicon sequence data) and multivariate Gaussian data (e.g. metabolomics data on the log-scale).

For samples *j* ∈ {1, …, *N*} we denote by *Y*_*j*_ the observed *D*_1_ -dimensional vector of sequence counts, *Z*_*j*_ the standardized (i.e. Z-transformed) and log_10_ -transformed *P* -dimensional vector of observed metabolite abundances, and *X*_*j*_ a *Q* -dimensional vector of covariates. Using this notation, the *orthus* likelihood model is given by

1 $$\begin{array}{l}Y_j \sim {{{\mathrm{Multinomial}}}}\left( {\pi _j} \right)\\ \pi _j = \phi ^{ - 1}\left( {\eta _j} \right)\\ \left[ {\begin{array}{*{20}{c}} {\eta _j^T} \\ {Z_j^T} \end{array}} \right]^T \sim N\left( {{{\Lambda }}X_j,{{\Sigma }}} \right)\end{array}$$

with priors ${{\Lambda }} \sim N({{\Theta }},{{\Sigma }},{{\Gamma }})$ and ${{\Sigma }} \sim$*Inverse Wishart* $\left( {{{\Xi }},v} \right)$ and with $\phi ^{ - 1}$ denoting the inverse additive log-ratio (ALR) transform with respect to the *D* -th taxa^77^. Of note, the ultimate inference is invariant to the chosen ALR transform. This represents a joint linear model over the latent relative abundances of microbial taxa and metabolite abundances. For computational scalability this model was inferred using the multinomial-Dirichlet Bootstrap approximation to the marginal posterior density *p* (π | *Y*) that is available in *fido*. The multinomial-Dirichlet bootstrap approximates the true marginal posterior density using the posterior of a Bayesian multinomial-Dirichlet model centred at the *maximum a posteriori* (*map*) estimate of π. In brief, this is accomplished as follows: for each sample *j*, the marginal posterior distribution *p* (π_*j*_ | *Y*) is approximated as the posterior of a Bayesian multinomial Dirichlet model $p(\tilde \pi \mid \tilde Y)$ where $\tilde Y = \pi _j^{map}\mathop {\sum}\nolimits_{i = 1}^D {Y_{ij}}$. Here, the Dirichlet parameters α_*j*_ were all taken to be 0.5; this can be thought of as a probabilistic equivalent to using a pseudo-count of 0.5 yet also producing quantified uncertainty due to multivariate counting. The prior parameters were chosen as

2 $${{\Theta }} = 0_{([D - 1 + P] \times Q)},{{\Gamma }} = I_q$$

and *v* = *D* + *P* + 9. Finally, we set the prior

3 $${{\Xi }} = (v - D - P)\,{\it{BlockDiagonal}}\left( {GG^T,I_P} \right)$$

where *G* is the (*D* − 1) × *D* ALR contrast matrix given by $G = \left[ {I_{D - 1} - 1} \right]$. This choice of Θ, Γ, ${{\Xi }}$, and *v* represents the weak prior belief that the correlation between the absolute abundance of taxa is, on average, small. This prior is closely related to the sparse penalization used by SparCC^78^. Using this model, priors, and inference, we sampled 2000 independent samples from the posterior distribution *p* (Λ, Σ | *Y*, *Z*, *X*).

Variable selection for these metabolite feature/microbiota correlation models was performed as follows: we selected the relevant variables based on multivariate analyses of the communities’ Bray-Curtis dissimilarity measure using PERMANOVA, a semi-parametric approach that does not assume multivariate normality. These analyses were performed separately for the within-Controls and within-PD groups. First, all clinical and technical variables of interest were analysed on their own in a univariable model (i.e. a model with a single explanatory variable). Given that the amount of variance explained in microbiome models is always very low, the choice of variables at this step was based solely on achieving statistical significance equal to or lower than 0.05. The variables that passed this alpha threshold of significance were then combined into a multivariable model (i.e. a model with more than one explanatory variable) using marginal testing. Those that retained significance at 0.05 or less in this full model were considered for the Bayesian covariance models. Of the latter, only those variables in common between the metabolite-based variable selection (see section iii above) and the microbiota-based variable selection were used for the Bayesian covariance models. Thus, for the three within-Parkinson’s covariance models (i.e. using only PD subjects at three taxonomic levels), the models were adjusted for COMT inhibitor medication use. For the three within-Controls covariance models we used intercept-only models.

The matrix Σ represents a ([*D* − 1] + *Q*) × ([*D* − 1] + *Q*) covariance matrix encoding all possible covariances between ALR coordinates and metabolites. For model interpretation and inference, each posterior sample of the upper (*D* − 1) × *Q* submatrix was transformed to a *D* × *Q* matrix representing the covariance between microbial composition (now represented with respect to centred log-ratio coordinates, or CLR) and metabolite abundances. Covariances were transformed to correlations using the function *cov2cor* in the R programming language. For the purposes of this study, we considered only those correlations that had a posterior mean equal to or larger than 0.3 and that had a 95% credible region not including zero. Conditioned on our chosen priors, this decision boundary can be thought of as limiting our results to correlations which we believe (with at least 95% certainty) are non-zero.

Covariance modelling was performed for bacterial genus, family, and phylum levels, using only metabolite abundance data at “Peak ID” level. This means that, although several of their corresponding MSI level 3 putative identifications were nominally the same, these were not merged before analysis, because they have different retention times and there is non-negligible uncertainty in their identification. After the correlations were calculated, we broadly assigned metabolite class information to these metabolites for Table 4 and Table 2 to aid in interpretation. These class assignments were then used to produce the Cytoscape^79^ network visualisations (v.3.8.0), both because classes simplify the networks and because they are more plausible than the putative MSI level 3 identifications. These classes were assigned by searching each putative identification of a metabolite feature against the Human Metabolome Database^16^ (HMDB) and the Kyoto Encyclopedia of Genes and Genomes^80^ (KEGG) entry (Table 6).

**Table 6:** Key to Figures 1 and 2.

Metabolite class key	Metabolite feature class	Metabolite class key	Metabolite feature class
AA	Amino acid	GL	Glycerolipid
AC	Acylcarnitines	GuD	Glucose derivative
AF	Antifungal	GPL	Glycerophospholipid
Al	Alkane	H	Hexoses
Ar	Arylamine	NT	Neurotoxin
ArC	Artificial chemical	OAAD	Organic acids and derivatives
Az	Azoles	OOC	Organooxygen compounds
C	Chemical	OS	Oligosaccharides
CA	Carboxylic acid	PF	Pyranoflavonoids
CAAD	Carboxylic acid and derivatives	PL	Prenol lipids
DA	Dicarboxylic acid	PN	Purine nucleosides
DODM	Drug or drug metabolite	PSL	Phosphosphingolipids
DOVA2	Derivative of Vitamin A2	SAD	Steroid and derivatives
EC	Epigallocatechins	SD	Sorbitol derivative
F	Flavonoids	SHC	Saturated hydrocarbon
FA	Fatty acyls	SL	Sphingolipid
FAA	Fatty acid amide	StL	Sterol lipid
FAG	Fatty acyl glycoside	TAD	Tyrosine and derivatives
FFA	Furanoic fatty acids	VDM	Vitamin D metabolite
GD	Galactose derivative	VKD	Vitamin K derivative

Genus key	Genus	Genus key	Genus
Acid	Acidaminococcus	Hold	Holdemanella
Akke	Akkermansia	Howa	Howardella
Allo	Alloprevotella	Inte	Intestinimonas
AnaeS	Anaerostipes	Kleb	Klebsiella
AnaeT	Anaerotruncus	Lach	Lachnospira
Asac	Asaccharobacter	Lact	Lactobacillus
Aste	Asteroleplasma	Mega	Megasphaera
Barn	Barnesiella	Odor	Odoribacter
Bifi	Bifidobacterium	Para	Parasutterella
Buty	Butyrivibrio	ParaP	Paraprevotella
Cate	Catenibacterium	Pept	Peptococcus
Cloa	Cloacibacillus	Phas	Phascolarctobacterium
Clos	Clostridium_sensu_stricto	Prev	Prevotella
ClosXIVb	Clostridium_XlVb	Porp	Porphyromonas
ClosXVIII	Clostridium_XVIII	Pseu	Pseudoflavonifractor
Coll	Collinsella	Romb	Romboutsia
Copr	Coprobacter	Rose	Roseburia
Desu	Desulfovibrio	Rumi	Ruminococcus
Dial	Dialister	Rumi2	Ruminococcus2
Dore	Dorea	Sene	Senegalimassilia
E/S	Escherichia/Shigella	Stre	Streptococcus
Eise	Eisenbergiella	Spor	Sporobacter
Euba	Eubacterium	Succ	Succiniclasticum
Faec	Faecalicoccus	Sutt	Sutterella
Flav	Flavonifractor	Turi	Turicibacter
Gemm	Gemmiger	Veil	Veillonella
Haem	Haemophilus	Vict	Victivallis

### Reporting summary

Further information on research design is available in the Nature Research Reporting Summary linked to this article.

## Supplementary information

Reporting Summary

Supplementary file

R script for Control samples

R script for PD samples

R script for utilities

## Data Availability

The 16S rRNA gene sequence raw data is available at the European Nucleotide Archive, with the accession number PRJEB27564. The metabolomics data is available at MetaboLights, with the accession number MTBLS4332. The clinical data is also available upon direct request from the corresponding authors of the present article. This is due to European patient confidentiality laws, and may require signing a Data Usage Agreement, depending on the specifics of the request.

## Code availability

Code for the bacterial taxa-metabolite correlation analyses is available as R scripts’ files (Supplementary files “Metabolomics.SERVER.SCRIPT.PD_ONLY.Selected.CORR.final” and “Metabolomics.SERVER.SCRIPT.CONTROLS_ONLY.Selected.CORR.final”). For metabolomics-only data analysis, deconvolution R scripts using XCMS and eRah, Matlab code for Partial Least Squares Discriminant Analysis (PLS-DA), R code for metabolite correlations, and Python code for Support Vector Machine (SVM) are available in the GitHub repository at github.com/drupadt/.
