# Supplementary material for: Multiomics implicate gut microbiota in altered lipid and energy metabolism in Parkinson’s disease
Source: NPJ Parkinsons Dis. 2022 Apr 11;8:39. doi: 10.1038/s41531-022-00300-3 (PMC9001728; doi:10.1038/s41531-022-00300-3)
Supplement: Supplementary file 1 — Reporting Summary [file 41531_2022_300_MOESM1_ESM.pdf]

nature portfolio

Corresponding author(s): Filip Scheperjans

Last updated by author(s): 2021/12/03

Reporting Summary

Nature Portfolio wishes to improve the reproducibility of the work that we publish. This form provides structure for consistency and transparency in reporting. For further information on Nature Portfolio policies, see our Editorial Policies and the Editorial Policy Checklist.

Statistics

For all statistical analyses, confirm that the following items are present in the figure legend, table legend, main text, or Methods section.

n/a	Confirmed	
☐	☒	The exact sample size (n) for each experimental group/condition, given as a discrete number and unit of measurement
☐	☒	A statement on whether measurements were taken from distinct samples or whether the same sample was measured repeatedly
☐	☒	The statistical test(s) used AND whether they are one- or two-sided<br>*Only common tests should be described solely by name; describe more complex techniques in the Methods section.*
☐	☒	A description of all covariates tested
☐	☒	A description of any assumptions or corrections, such as tests of normality and adjustment for multiple comparisons
☐	☒	A full description of the statistical parameters including central tendency (e.g. means) or other basic estimates (e.g. regression coefficient) AND variation (e.g. standard deviation) or associated estimates of uncertainty (e.g. confidence intervals)
☒	☐	For null hypothesis testing, the test statistic (e.g. F, t, r) with confidence intervals, effect sizes, degrees of freedom and P value noted<br>*Give P values as exact values whenever suitable.*
☐	☒	For Bayesian analysis, information on the choice of priors and Markov chain Monte Carlo settings
☒	☐	For hierarchical and complex designs, identification of the appropriate level for tests and full reporting of outcomes
☐	☒	Estimates of effect sizes (e.g. Cohen's d, Pearson's r), indicating how they were calculated

Our web collection on statistics for biologists contains articles on many of the points above.

Software and code

Policy information about availability of computer code

Data collection	No software was used for data collection.
Data analysis	Mummichog(v.1.0.9)<br>R Statistical Programming Software (v.3.6.0)<br>Cytoscape (v.3.8.0)<br>Matlab (v.9.10)

For manuscripts utilizing custom algorithms or software that are central to the research but not yet described in published literature, software must be made available to editors and reviewers. We strongly encourage code deposition in a community repository (e.g. GitHub). See the Nature Portfolio guidelines for submitting code & software for further information.

Data

Policy information about availability of data

All manuscripts must include a data availability statement. This statement should provide the following information, where applicable:

- Accession codes, unique identifiers, or web links for publicly available datasets
- A description of any restrictions on data availability
- For clinical datasets or third party data, please ensure that the statement adheres to our policy

The 16S rRNA gene sequence abundance raw data is available from Aho et al. (2019), including accession codes. The metabolomics data will be hosted on MetaboLights (https://www.ebi.ac.uk/metabolights/index) and also on The University of Manchester servers. It will also be made available upon reasonable request. The clinical data necessary to reproduce the analyses is also available upon request due to European subject confidentiality laws.
Code for the bacterial taxa-metabolite correlation analyses is available as R scripts' files (supplementary files

"Metabolomics.SERVER.SCRIPT.PD_ONLY.Selected.CORR.final" and "Metabolomics.SERVER.SCRIPT.CONTROLS_ONLY.Selected.CORR.final"). For metabolomics-only data analysis, all code is available in the GitHub repository at github.com/drupadt/

Field-specific reporting

Please select the one below that is the best fit for your research. If you are not sure, read the appropriate sections before making your selection.

☒ Life sciences ☐ Behavioural & social sciences ☐ Ecological, evolutionary & environmental sciences

For a reference copy of the document with all sections, see nature.com/documents/nr-reporting-summary-flat.pdf

Life sciences study design

All studies must disclose on these points even when the disclosure is negative.

Sample size	The data used was the same as in a previous study (Aho et al. 2019). These studies are exploratory and do not have a specific target for effect sizes. Therefore, no power analysis was performed to predetermine sample size. The study has a larger sample size than the average of studies in this topic/field and therefore should be sufficient for it's exploratory objectives.
Data exclusions	The dataset is the same as inn Aho et al. (2019) given that the same data was used for the present study minus 4 samples that were excluded from the present analysis during quality control of the metabolomics data. These samples are identified in the present manuscript.
Replication	Due to the observational nature of the study and its clinical nature (a set of patients that are being followed for their condition), no replication is possible in practice.
Randomization	Not relevant for the study given that it's not an experimental study. Relevant covariates were analyzed and adjusted for in the statistical models using common practices for model variable selection in data-driven studies based on high-throughput 'omics methods. Details are provided in the manuscript.
Blinding	It's a case-control observational study, therefore blinding is not relevant.

Reporting for specific materials, systems and methods

We require information from authors about some types of materials, experimental systems and methods used in many studies. Here, indicate whether each material, system or method listed is relevant to your study. If you are not sure if a list item applies to your research, read the appropriate section before selecting a response.

Materials & experimental systems

n/a	Involved in the study	
☒	☐	Antibodies
☒	☐	Eukaryotic cell lines
☒	☐	Palaeontology and archaeology
☒	☐	Animals and other organisms
☒	☐	Human research participants
☒	☐	Clinical data
☒	☐	Dual use research of concern

Methods

n/a	Involved in the study	
☒	☐	ChIP-seq
☒	☐	Flow cytometry
☒	☐	MRI-based neuroimaging
