# Supplementary material for: Multiomics implicate gut microbiota in altered lipid and energy metabolism in Parkinson’s disease
Source: NPJ Parkinsons Dis. 2022 Apr 11;8:39. doi: 10.1038/s41531-022-00300-3 (PMC9001728; doi:10.1038/s41531-022-00300-3)

Supplementary population data table

	Present study				Aho et al. study			
Variable	Control subjects (% (n) / mean ± SD / median [IQR])	Parkinson's patients (% (n) / mean ± SD / median [IQR])	*p*-value		Control subjects (% (n) / mean ± SD / median [IQR])	Parkinson's patients (% (n) / mean ± SD / median [IQR])	*p*-value	Test
**n**	61	63			64	64		
**Age (at second stool collection)**	66.56 ± 6.94	67.38 ± 5.54	0.468		66.53 ± 6.89	67.33 ± 5.51	0.471	Student's *t*
**BMI**	26.92 [24.24-28.63]	27.26 [24.06-30.09]	0.444		26.94 [24.32-28.64]	27.24 [23.95-30.08]	0.572	Wilcoxon Rank Sum
**Sex (% males)**	49.18(30)	50.79(32)	1.000		50(32)	51.56(33)	1.000	Fisher's Exact
**History of TIA or ischemic stroke**	39.34(24)	7.94(5)	***<0.001***		37.5(24)	7.81(5)	***<0.001***	Fisher's Exact
**Medication: calcium channel blockers**	19.67(12)	6.35(4)	**0.033**		20.31(13)	6.25(4)	**0.035**	Fisher's Exact
**Medication: statins**	50.82(31)	20.63(13)	***<0.001***		50(32)	20.31(13)	***<0.001***	Fisher's Exact
**Medication: warfarin**	14.75(9)	4.76(3)	0.073		15.62(10)	4.69(3)	0.076	Fisher's Exact
**NMSS total**	6 [3-11]	40 [20-59.5]	***<0.001***		6 [2-10.25]	40 [19.75-58.75]	***<0.001***	Wilcoxon Rank Sum
**Rome III constipation-defecation score (sum of items 9–15)**	2 [0-3]	8 [2.5-11]	***<0.001***		2 [0-3]	8 [2.75-11]	***<0.001***	Wilcoxon Rank Sum
**Rome III IBS criteria fulfilled**	8.2(5)	36.51(23)	***<0.001***		7.81(5)	35.94(23)	***<0.001***	Fisher's Exact

Table legend: Statistically significant p-values are marked in bold italic font. SD: Standard Deviation, IQR: Interquartile Range, BMI: Body Mass Index, TIA: Transient Ischemic Attack, NMSS: Non-Motor Symptoms Scale, IBS: Irritable Bowel Syndrome.

This table corresponds to Table 3 in the Aho et al. 2019 study, showing the difference in our study after removal of 4 samples.

Supplementary Table 1

Bacterial Genus	Metabolite Peak ID	Metabolite MSI 3 ID	Class	p2.5	Mean	p97.5
Eisenbergiella	X144	1alpha,24,25,28-tetrahydroxyergocalciferol	Vitamin D metabolite	-0.594904730555633	**-0.40652180518621**	-0.197161171365051
Eisenbergiella	X420	PC(16:1(9Z)/0:0)	Glycerophospholipid	-0.590049595036504	**-0.401178236329287**	-0.194762397452913
Erysipelotrichaceae_(unclassified_genus)	X6877	5-Methyltetrahydropteroyltri-L-glutamate	Steroid and derivaties	-0.583304481560543	**-0.398577947413559**	-0.192169004088139
Butyrivibrio	X439	PI(16:0/20:1(11Z))	Glycerophospholipid	-0.591834178602526	**-0.39479972613378**	-0.163652186776382
Erysipelotrichaceae_(unclassified_genus)	X7003	MGDG(20:5(5Z,8Z,11Z,14Z,17Z)/18:3(9Z,12Z,15Z))	Glycerolipid	-0.582861891933091	**-0.393153044806285**	-0.174362964119626
Eisenbergiella	X32	Jolkinol B	Chemical	-0.58854601445467	**-0.39298138499959**	-0.179210869641538
Eisenbergiella	X500	Butyrylcarnitine	Fatty acyls	-0.577034951222831	**-0.390539275875657**	-0.188453225000917
Mollicutes_(unclassified_order)	X170	PA(P-18:0/17:2(9Z,12Z))	Glycerophospholipid	-0.618032107944109	**-0.38710179175322**	-0.138254993073495
Eisenbergiella	X370	3-octadecylenic acid	Fatty acyls	-0.574571994898339	**-0.383614654024466**	-0.177723651792172
Eisenbergiella	X406	SM(d16:1/22:0)	Sphingolipid	-0.56437022751873	**-0.383032483993568**	-0.164412330903548
Butyrivibrio	X204	2,3-epoxyphylloquinone	Vitamin K derivative	-0.578274465271035	**-0.381431665520996**	-0.152528063193689
Eisenbergiella	X497	SM(d17:1/24:1)	Sphingolipid	-0.572731533290932	**-0.380014380850253**	-0.167720875079737
Eisenbergiella	X498	SM(d18:2/21:0)	Sphingolipid	-0.571333007001349	**-0.378929355378666**	-0.182221558332317
Burkholderiales_(unclassified_family)	X6288	Sphingosine-1-phosphate	Phosphosphingolipids	-0.556932272549387	**-0.376498034841568**	-0.160125749537363
Mollicutes_(unclassified_order)	X337	C17 sphingosine-1-phosphocholine	Sphingolipid	-0.572712494577712	**-0.371176432569008**	-0.136529689502371
Mollicutes_(unclassified_order)	X320	11-cis-Dehydroretinal	Derivative of Vitamin A2	-0.566852609138309	**-0.369097154084496**	-0.126306411458034
Acidaminococcaceae_(unclassified_genus)	X7280	Heptadecane	Alkane	-0.554100929830109	**-0.362582144432628**	-0.143860825094098
Eisenbergiella	X362	PC(22:4(7Z,10Z,13Z,16Z)/0:0)	Glycerophospholipid	-0.559960067425361	**-0.359229214114873**	-0.143436765956979
Mollicutes_(unclassified_order)	X13	Leukotriene D5	Organooxygen compounds	-0.565847576479659	**-0.357715877339998**	-0.115221444774818
Akkermansia	X7038	PE(20:2(11Z,14Z)/22:5(4Z,7Z,10Z,13Z,16Z))	Glycerophospholipid	-0.545386123859077	**-0.357246042268429**	-0.135044482332691
Senegalimassilia	X474	Acevaltrate	Carboxylic acid	-0.55635833675803	**-0.353511941531634**	-0.134139115103469
Phascolarctobacterium	X311	Sphingofungin A	Antifungal	-0.542613062598836	**-0.352469431693504**	-0.132485334874569
Alloprevotella	X6619	FAHFA(18:1(9Z)/13-O-18:0)	Fatty acyls	-0.555938015656238	**-0.350918046364383**	-0.138316779110083
Senegalimassilia	X153	Withaperuvin B	Steroid and derivaties	-0.54071148071996	**-0.347664903966874**	-0.126442570590078
Victivallis	X7206	N-formyl-methionine	Carboxylic acid and derivatives	-0.533155368915443	**-0.345190131120815**	-0.134804833373678
Erysipelotrichaceae_(unclassified_genus)	X6916	PC(P-16:0/18:4(6Z,9Z,12Z,15Z))	Glycerophospholipid	-0.53394386498124	**-0.342612245076685**	-0.130468445764506
Clostridia_(unclassified_order)	X429	PC(16:0/18:1(6Z))	Glycerophospholipid	-0.527828539555013	**-0.342515922826395**	-0.130738505277472
Burkholderiales_(unclassified_family)	X7263	2-phenyl-glycine	Chemical	-0.529116464266689	**-0.342238852431417**	-0.129972335402508
Holdemanella	X7170	Tricosane	Saturated hydrocarbon	-0.536490290399345	**-0.342096240023225**	-0.112853827966516
Anaerostipes	X6174	N2,N2-Dimethylguanosine	Purine nucleosides	-0.527925494021111	**-0.341619709228958**	-0.123847338599257
Eisenbergiella	X490	PA(O-20:0/13:0)	Glycerophospholipid	-0.547169697724504	**-0.341616701125196**	-0.131116134099565
Eisenbergiella	X327	Anandamide (20:5, n-3)	Fatty acid amide	-0.535989818181172	**-0.339975918297603**	-0.124502842596333
Rikenellaceae_(unclassified_genus)	X393	Propionylcarnitine	Fatty acyls	-0.534295440740139	**-0.33674839884672**	-0.122055672015904
Clostridium_sensu_stricto	X7236	4-hydroxy-, trans-proline	Carboxylic acid and derivatives	-0.526415260073527	**-0.33564392912765**	-0.13188475031659
Roseburia	X499	3-Deoxyvitamin D3	Sterol lipid	-0.524913858708013	**-0.334817291073649**	-0.115874540368365
Victivallis	X430	PC(P-20:0/18:3(6Z,9Z,12Z))	Glycerophospholipid	-0.523663044121178	**-0.334670887580696**	-0.124219850005
Mollicutes_(unclassified_order)	X67	4-O-alpha-Cadinylangolensin	Flavonoids	-0.55080213188873	**-0.334291415319941**	-0.0876087746685072

Desulfovibrionaceae_(unclassified_genus)	X363	3-carboxy-4-methyl-5-pentyl-2-furanpropanoic acid	Furanoic fatty acids	-0.537709439563817	**-0.332670414207464**	-0.0967895612483911
Phascolarctobacterium	X498	SM(d18:2/21:0)	Sphingolipid	-0.524713998117744	**-0.331931303471281**	-0.120450026402114
Senegalimassilia	X263	Epigallocatechin 3-O-caffeate	Epigallocatechins	-0.527042299753342	**-0.33165645868797**	-0.110283544728985
Eisenbergiella	X399	PE(O-20:0/22:6(4Z,7Z,10Z,13Z,16Z,19Z))	Glycerophospholipid	-0.536027914317947	**-0.331220087033203**	-0.108174115856349
Bacteroidetes_(unclassified_class)	X474	Acevaltrate	Carboxylic acid	-0.525962713093897	**-0.331182613375861**	-0.110577773123247
Prevotella	X7253	Proline	Carboxylic acid and derivatives	-0.514614613618309	**-0.330740423935374**	-0.118574844273797
Clostridia_(unclassified_order)	X276	SM(d18:0/24:0)	Sphingolipid	-0.526313886135935	**-0.330689089345877**	-0.113590352192214
Asteroleplasma	X6319	Galbanic acid	Prenol lipids	-0.534095729041882	**-0.330330591625469**	-0.100270923722784
Bacteroidetes_(unclassified_class)	X518	PC(18:4(6Z,9Z,12Z,15Z)/18:1(11Z))	Glycerophospholipid	-0.517314773554419	**-0.328952490202561**	-0.113837573842791
Bacteroidetes_(unclassified_class)	X263	Epigallocatechin 3-O-caffeate	Epigallocatechins	-0.522374344271754	**-0.328190370930582**	-0.102397896410514
Butyricimonas	X7038	PE(20:2(11Z,14Z)/22:5(4Z,7Z,10Z,13Z,16Z))	Glycerophospholipid	-0.51869137814875	**-0.326553730794986**	-0.101058090012426
Erysipelotrichaceae_(unclassified_genus)	X6860	PE(16:0/P-18:1(11Z))	Glycerophospholipid	-0.528958033664845	**-0.326541757014699**	-0.106230194150157
Asteroleplasma	X276	SM(d18:0/24:0)	Sphingolipid	-0.538981153568736	**-0.326201762664493**	-0.0947572990442303
Bacteroidetes_(unclassified_class)	X153	Withaperuvin B	Steroid and derivaties	-0.518344986579125	**-0.325920362041729**	-0.107387939753598
Mollicutes_(unclassified_order)	X295	Glycoursodeoxycholic acid	Steroid and derivaties	-0.549381295708042	**-0.325867745292625**	-0.0686745136459645
Cloacibacillus	X7207	Maltotriose	Oligosaccharides	-0.532382796408269	**-0.325373699431123**	-0.0795954203814991
Lactobacillus	X6092	3,4-dimethyl-5-carboxyethyl-2-furanpentanoic acid	Furanoic fatty acids	-0.526973591545645	**-0.324640156202657**	-0.0991345817959156
Bacteroidetes_(unclassified_class)	X327	Anandamide (20:5, n-3)	Fatty acid amide	-0.530826282710867	**-0.323439395724892**	-0.0901835859912043
Rhodospirillaceae_(unclassified_genus)	X7233	Pentadecane	Alkane	-0.522364887146834	**-0.323209997136109**	-0.102819543463832
Senegalimassilia	X264	6-Keto-decanoylcarnitine	Fatty acyls	-0.534230149655858	**-0.321885131244057**	-0.0965177486824383
Cloacibacillus	X348	Fuconic acid	Chemical	-0.537025425268834	**-0.321433591235351**	-0.0775515463352789
Bacteroidetes_(unclassified_class)	X459	PC(P-18:0/20:5(5Z,8Z,11Z,14Z,17Z))	Glycerophospholipid	-0.523392869088573	**-0.321395172919372**	-0.0960700911094945
Turicibacter	X6277	Veranisatin C	Prenol lipids	-0.521319567791645	**-0.32081125120911**	-0.0790287847174881
Alloprevotella	X6065	Palmitelaidic acid	Fatty acyls	-0.529288009098978	**-0.319195612316097**	-0.0920576535024359
Cloacibacillus	X369	PE-Cer(d15:2(4E,6E)/22:0(2OH))	Glycerophospholipid	-0.517102355279435	**-0.316124891436118**	-0.0943087991241784
Prevotella	X7287	Tartronic acid	Dicarboxylic acid	-0.502458545092644	**-0.314799894617226**	-0.106630446673331
Clostridia_(unclassified_order)	X130	7,3'-Dihydroxy-4'-methoxy-8-methylflavan	Flavonoids	-0.509832479917362	**-0.314478518588354**	-0.096486448650886
Holdemanella	X7206	N-formyl-methionine	Carboxylic acid and derivatives	-0.539922675884564	**-0.312975417136969**	-0.071919716188211
Alphaproteobacteria_(unclassified_order)	X511	PC(18:3(9Z,12Z,15Z)/0:0)	Glycerophospholipid	-0.522166795025347	**-0.309969163721542**	-0.0792802910613255
Rhodospirillales_(unclassified_family)	X361	PC(18:3(9Z,12Z,15Z)/0:0)	Glycerophospholipid	-0.518530631244732	**-0.308518565542603**	-0.0950364505800571
Burkholderiales_(unclassified_family)	X6564	PS(19:0/0:0)	Glycerophospholipid	-0.509354549008399	**-0.307326869706996**	-0.0815715247269464
Butyrivibrio	X363	3-carboxy-4-methyl-5-pentyl-2-furanpropanoic acid	Furanoic fatty acids	-0.520113337943551	**-0.306739691670229**	-0.0589861624871708
Prevotella	X7206	N-formyl-methionine	Carboxylic acid and derivatives	-0.495674396431823	**-0.30627243465172**	-0.0928631381176446
Mollicutes_(unclassified_order)	X439	PI(16:0/20:1(11Z))	Glycerophospholipid	-0.529028173909865	**-0.305136267759885**	-0.0660485568304778
Parasutterella	X6860	PE(16:0/P-18:1(11Z))	Glycerophospholipid	-0.498955011817565	**-0.304743674251121**	-0.0661259825961377
Eisenbergiella	X458	20:2-Glc-Campesterol	Sterol lipid	-0.511430315411114	**-0.304665030458932**	-0.0908181754660633
Bacteria_(unclassified_phylum)	X6277	Veranisatin C	Prenol lipids	-0.49968584705984	**-0.304105694371105**	-0.0915762480623126
Eisenbergiella	X331	Cholesterol sulfate	Steroid and derivaties	-0.504884506937036	**-0.303591006656493**	-0.0889835508441905
Senegalimassilia	X191	Sphinganine-phosphate	Phosphosphingolipids	-0.505371748377278	**-0.303355690039698**	-0.0822343691801208

Barnesiella	X24	Cycloheterophyllin	Pyranoflavonoids	-0.49432758310008	**-0.301567313527808**	-0.0879787375925506
Haemophilus	X7236	4-hydroxy-, trans-proline	Carboxylic acid and derivatives	-0.507596703040233	**-0.301025459513394**	-0.0773154355546684
Rhodospirillales_(unclassified_family)	X7183	Beta Alanine	Carboxylic acid	-0.50572787608732	**-0.30054290123855**	-0.0754202043096277
Cloacibacillus	X443	GlcCer(d18:1(8E)/21:0(2OH[R]))	Sphingolipid	-0.514167614600815	**-0.300330595242512**	-0.0714418794960905
Desulfovibrionaceae_(unclassified_genus)	X115	3-hydroxybutyrylcarnitine	Acylcarnitines	0.0625161687390715	**0.300990456150484**	0.522742919629485
Lachnospira	X393	Propionylcarnitine	Fatty acyls	0.0990968443948681	**0.302583886588122**	0.499633491274496
Peptococcus	X361	3,4-dimethyl-5-carboxyethyl-2-furanhexanoic acid	Furanoic fatty acids	0.0591831764241016	**0.304048156121803**	0.515294022883387
Bacteroidales_(unclassified_family)	X7003	MGDG(20:5(5Z,8Z,11Z,14Z,17Z)/18:3(9Z,12Z,15Z))	Glycerolipid	0.0572296553340556	**0.304201991036802**	0.521706162553936
Coprobacter	X7278	Tetradecane	Saturated hydrocarbon	0.0667972642262634	**0.304410675085908**	0.536465294353887
Parasutterella	X144	1alpha,24,25,28-tetrahydroxyergocalciferol	Vitamin D metabolite	0.0750064086319753	**0.304889824301377**	0.500821697252344
Clostridium_XVIII	X7205	Serine	Amino acid	0.0866818925886348	**0.304956118473247**	0.504692138106614
Clostridium_XVIII	X7183	Beta Alanine	Carboxylic acid	0.0758042016358035	**0.305604328902766**	0.508027602172656
Clostridia_(unclassified_order)	X7263	2-phenyl-glycine	Chemical	0.0737410402234729	**0.305883502883862**	0.515511691839944
Collinsella	X24	Cycloheterophyllin	Pyranoflavonoids	0.0738458628902783	**0.306345737464217**	0.504758178533021
Clostridium_XlVb	X151	PE(18:4(6Z,9Z,12Z,15Z)/18:1(9Z))	Glycerophospholipid	0.0970041326996784	**0.306412003484158**	0.505538534440247
Sporobacter	X420	PC(16:1(9Z)/0:0)	Glycerophospholipid	0.0637195103804183	**0.306460819093719**	0.516850745479486
Ruminococcus2	X511	PC(18:3(9Z,12Z,15Z)/0:0)	Glycerophospholipid	0.0914424525208464	**0.306612633226351**	0.51043201434131
Roseburia	X423	PC(P-20:0/18:2(9Z,12Z))	Glycerophospholipid	0.10335597701467	**0.30697504343341**	0.497008962569915
Klebsiella	X467	PE-Cer(d15:1(4E)/18:0)	Glycerophospholipid	0.0618240625309383	**0.307002727158631**	0.517146562907435
Erysipelotrichaceae_(unclassified_genus)	X7271	Decane	Alkane	0.0837019928160431	**0.307073705710145**	0.510589086560667
Lachnospira	X509	PI(O-16:0/13:0)	Glycerophospholipid	0.0889227945516533	**0.307386151510497**	0.495633219680987
Erysipelotrichaceae_(unclassified_genus)	X7038	PE(20:2(11Z,14Z)/22:5(4Z,7Z,10Z,13Z,16Z))	Glycerophospholipid	0.0711565478822224	**0.308320536928501**	0.514744882522281
Erysipelotrichaceae_(unclassified_genus)	X7170	Tricosane	Saturated hydrocarbon	0.0579224856148206	**0.308639725713695**	0.542346005141904
Clostridia_(unclassified_order)	X7241	3,4-dimethyl-aniline	Arylamine	0.0686584563864517	**0.308729030595644**	0.509871477732799
Sporobacter	X406	SM(d16:1/22:0)	Sphingolipid	0.0633796683578089	**0.309206051135729**	0.519667055328427
Mollicutes_(unclassified_order)	X406	SM(d16:1/22:0)	Sphingolipid	0.050712993570625	**0.309601106657555**	0.537521371161741
Flavonifractor	X7163	2-amino-2-deoxy-glucose	Hexoses	0.094811552215851	**0.309727191553749**	0.503555646696395
Sporobacter	X429	PC(16:0/18:1(6Z))	Glycerophospholipid	0.0639374571307061	**0.309813021230914**	0.538787274742837
Butyricimonas	X6092	3,4-dimethyl-5-carboxyethyl-2-furanpentanoic acid	Furanoic fatty acids	0.0917358669313067	**0.310687061266725**	0.506878722220818
Klebsiella	X201	Butyrylcarnitine	Fatty acyls	0.0659362694326487	**0.310990149271584**	0.529446032340158
Desulfovibrionaceae_(unclassified_genus)	X502	PE(P-18:0/20:5(5Z,8Z,11Z,14Z,17Z))	Glycerophospholipid	0.0750275344660234	**0.311331811268945**	0.521753688965622
Clostridium_XVIII	X7264	N-acetyl-cysteine	Drug or drug metabolite	0.0875045355936598	**0.312302694380579**	0.518312640963073
Flavonifractor	X7271	Decane	Alkane	0.0969912785626502	**0.312355477233976**	0.515651197306337
Mollicutes_(unclassified_order)	X420	PC(16:1(9Z)/0:0)	Glycerophospholipid	0.0439538911922634	**0.313083789719745**	0.534805522335418
Sporobacter	X369	PE-Cer(d15:2(4E,6E)/22:0(2OH))	Glycerophospholipid	0.0591060784725357	**0.313537574210635**	0.530524846743967
Clostridium_XVIII	X7175	Dodecane	Alkane	0.0838764361188573	**0.314629278969548**	0.517079079848663
Erysipelotrichaceae_(unclassified_genus)	X7160	Dodecane	Alkane	0.0766888243749567	**0.315097594286596**	0.512532243376286
Parasutterella	X502	PE(P-18:0/20:5(5Z,8Z,11Z,14Z,17Z))	Glycerophospholipid	0.0960740258354064	**0.315443540944807**	0.512897069044007
Desulfovibrionaceae_(unclassified_genus)	X287	Fuconic acid	Chemical	0.0748458638071618	**0.315463114306745**	0.536630664725985

Acidaminococcus	X13	Leukotriene D5	Organooxygen compounds	0.0650129034043268	**0.316729517222875**	0.544963067035238
Dialister	X370	3-octadecylenic acid	Fatty acyls	0.0932377310125915	**0.317136206926388**	0.509095210864907
Klebsiella	X264	6-Keto-decanoylcarnitine	Fatty acyls	0.0835274712416154	**0.317537066876645**	0.525868482893558
Mollicutes_(unclassified_order)	X331	Cholesterol sulfate	Steroid and derivaties	0.0682575036049729	**0.317624748370742**	0.535557710941031
Klebsiella	X153	Withaperuvin B	Steroid and derivaties	0.0830140870314013	**0.31879392511421**	0.531984785834624
Mollicutes_(unclassified_order)	X7003	MGDG(20:5(5Z,8Z,11Z,14Z,17Z)/18:3(9Z,12Z,15Z))	Glycerolipid	0.0665883446459043	**0.318889655479513**	0.549497603034508
Clostridium_XVIII	X7279	Proline	Carboxylic acid and derivatives	0.0957398265443772	**0.319462401788243**	0.523117778529502
Turicibacter	X327	Anandamide (20:5, n-3)	Fatty acid amide	0.0852340966431759	**0.320420788256926**	0.530970183883399
Parasutterella	X327	Anandamide (20:5, n-3)	Fatty acid amide	0.112137486298051	**0.320525667691508**	0.515528751965974
Sporobacter	X144	1alpha,24,25,28-tetrahydroxyergocalciferol	Vitamin D metabolite	0.0755762055390832	**0.322304217178077**	0.531549661431572
Sporobacter	X115	3-hydroxybutyrylcarnitine	Acylcarnitines	0.0719145995380156	**0.323232824615025**	0.528338934711071
Lactobacillus	X7038	PE(20:2(11Z,14Z)/22:5(4Z,7Z,10Z,13Z,16Z))	Glycerophospholipid	0.0916006474801668	**0.32596094686495**	0.529106008114934
Parasutterella	X70	(3S,5R,6S,7E,9x)-7-Megastigmene-3,6,9-triol 9-glucoside	Fatty acyl glycoside	0.107213726166998	**0.326074173020543**	0.51788785597031
Mollicutes_(unclassified_order)	X500	Butyrylcarnitine	Fatty acyls	0.0632624402914899	**0.329135326109636**	0.537727075635882
Erysipelotrichaceae_(unclassified_genus)	X7171	Pyroglutamic acid	Carboxylic acid and derivatives	0.0980016198938663	**0.329592583119952**	0.53667349567609
Roseburia	X370	3-octadecylenic acid	Fatty acyls	0.123131934535937	**0.329975706816786**	0.520826062039666
Akkermansia	X6564	PS(19:0/0:0)	Glycerophospholipid	0.105577468550035	**0.33049078787443**	0.530417856090504
Sutterella	X6564	PS(19:0/0:0)	Glycerophospholipid	0.113652337132084	**0.331613580461422**	0.532513860542978
Klebsiella	X263	Epigallocatechin 3-O-caffeate	Epigallocatechins	0.0953764603081059	**0.332236611527093**	0.539610878819517
Erysipelotrichaceae_(unclassified_genus)	X7207	Maltotriose	Oligosaccharides	0.103114102580036	**0.332553078034169**	0.538473286241019
Butyricimonas	X6752	NA	Chemical	0.116814913459216	**0.334411190766188**	0.536449478769506
Mollicutes_(unclassified_order)	X458	20:2-Glc-Campesterol	Sterol lipid	0.0922858352179215	**0.334579942078295**	0.555289129795808
Victivallis	X6092	3,4-dimethyl-5-carboxyethyl-2-furanpentanoic acid	Furanoic fatty acids	0.110797413375911	**0.335942536947665**	0.541279113653938
Parasutterella	X369	PE-Cer(d15:2(4E,6E)/22:0(2OH))	Glycerophospholipid	0.103821823085481	**0.336353171527692**	0.53369339283075
Collinsella	X75	Stearoyl tyrosine	Tyrosine and derivatives	0.118015287579403	**0.336809008229295**	0.524676017622479
Clostridium_XVIII	X7171	Pyroglutamic acid	Carboxylic acid and derivatives	0.132531176308518	**0.337006248459429**	0.536901940912086
Desulfovibrionaceae_(unclassified_genus)	X443	Pyroglutamic acid	Carboxylic acid and derivatives	0.100028724164545	**0.338468555144855**	0.541441053867378
Sutterella	X6565	Tetrahydroaldosterone-3-glucuronide	Steroid and derivaties	0.120965439606179	**0.338818854521163**	0.531237060182
Erysipelotrichaceae_(unclassified_genus)	X7205	Serine	Amino acid	0.106476955854361	**0.342392120135523**	0.550237605442965
Clostridium_XVIII	X7210	3-imidazole-lactic acid	Azoles	0.121837081670866	**0.342397957683075**	0.554940017964156
Parasutterella	X130	7,3'-Dihydroxy-4'-methoxy-8-methylflavan	Flavonoids	0.125181989559751	**0.343700869482914**	0.54594495624236
Turicibacter	X144	1alpha,24,25,28-tetrahydroxyergocalciferol	Vitamin D metabolite	0.103402600905048	**0.34434592487054**	0.552121815336555
Sutterella	X7081	PS(19:0/22:6(4Z,7Z,10Z,13Z,16Z,19Z))	Glycerophospholipid	0.114257851701079	**0.346364983441288**	0.542163411824419
Lachnospira	X7268	Heptadecane	Alkane	0.136698394564401	**0.349450836743494**	0.536342483635345
Flavonifractor	X7183	Beta Alanine	Carboxylic acid	0.145183892268797	**0.35419492034028**	0.531490453746933
Clostridium_XVIII	X361	3,4-dimethyl-5-carboxyethyl-2-furanhexanoic acid	Furanoic fatty acids	0.133148733239924	**0.355271912239964**	0.547815057124758
Erysipelotrichaceae_(unclassified_genus)	X7279	Proline	Carboxylic acid and derivatives	0.118417963115801	**0.356679827402127**	0.564783113295876
Butyricicoccus	X370	3-octadecylenic acid	Fatty acyls	0.147380208827847	**0.356816443912341**	0.549935801587768
Senegalimassilia	X6277	Veranisatin C	Prenol lipids	0.12019410169467	**0.357031754484729**	0.571223495780259

Mollicutes_(unclassified_order)	X32	Jolkinol B	Chemical	0.110747365103151	**0.357603556872304**	0.568019637795379
Sutterella	X6288	Sphingosine-1-phosphate	Phosphosphingolipids	0.137529225111949	**0.358436467834098**	0.555379958171547
Erysipelotrichaceae_(unclassified_genus)	X7246	4-hydroxy-, trans-proline	Carboxylic acid and derivatives	0.136887269843704	**0.361289011325999**	0.555047822112543
Desulfovibrionaceae_(unclassified_genus)	X276	SM(d18:0/24:0)	Sphingolipid	0.129039357983122	**0.362840363620044**	0.575987024467802
Bacteroidales_(unclassified_family)	X6564	PS(19:0/0:0)	Glycerophospholipid	0.127756146486835	**0.363950932649092**	0.576319213003623
Alloprevotella	X430	PC(P-20:0/18:3(6Z,9Z,12Z))	Glycerophospholipid	0.145115074477198	**0.369278229077602**	0.559462086255614
Eisenbergiella	X337	C17 sphingosine-1-phosphocholine	Sphingolipid	0.162932191408951	**0.372758013632929**	0.573669375790544
Sutterella	X7003	MGDG(20:5(5Z,8Z,11Z,14Z,17Z)/18:3(9Z,12Z,15Z))	Glycerolipid	0.169931122715248	**0.375632106969165**	0.558989489112285
Eisenbergiella	X463	PC(P-16:0/20:3(8Z,11Z,14Z))	Glycerophospholipid	0.16386399934615	**0.37780129226462**	0.566946210136583
Desulfovibrionaceae_(unclassified_genus)	X313	Iodovulone I	Chemical	0.157847690399611	**0.379079766648811**	0.572947461719347
Erysipelotrichaceae_(unclassified_genus)	X7175	Dodecane	Alkane	0.153744478253477	**0.379781418871219**	0.570118479812993
Dorea	X7195	Tridecane	Saturated hydrocarbon	0.161160110808418	**0.381936653761614**	0.571745135282486
Eisenbergiella	X439	PI(16:0/20:1(11Z))	Glycerophospholipid	0.179629191765196	**0.387188392243737**	0.571812727484654
Eisenbergiella	X243	PS(20:3(8Z,11Z,14Z)/0:0)	Glycerophospholipid	0.196626158916457	**0.39222282619147**	0.576378889104825
Coriobacteriaceae_(unclassified_genus)	X36	PG(16:1(9Z)/22:4(7Z,10Z,13Z,16Z))	Glycerophospholipid	0.195241216339468	**0.401804842547503**	0.578031110230967
Coriobacteriaceae_(unclassified_genus)	X75	Stearoyl tyrosine	Tyrosine and derivatives	0.205953878522268	**0.404097617730905**	0.580819198462608
Erysipelotrichaceae_(unclassified_genus)	X7253	Proline	Carboxylic acid and derivatives	0.194129599268985	**0.404196522730133**	0.592039935989841
Eisenbergiella	X320	11-cis-Dehydroretinal	Derivative of Vitamin A2	0.195844395332594	**0.404749580804787**	0.594734951900803
Erysipelotrichaceae_(unclassified_genus)	X7264	N-acetyl-cysteine	Drug or drug metabolite	0.176883582165947	**0.407883666713215**	0.601962081723418
Sutterella	X6916	PC(P-16:0/18:4(6Z,9Z,12Z,15Z))	Glycerophospholipid	0.192304925873428	**0.408591484113137**	0.59909927248987
Sutterella	X6860	PE(16:0/P-18:1(11Z))	Glycerophospholipid	0.242039033300524	**0.446350394986057**	0.624382275872088
Sutterella	X6877	5-Methyltetrahydropteroyltri-L-glutamate	Steroid and derivaties	0.230323689555212	**0.446490598758583**	0.624078268292112

NOTE: Unclassified taxa may contain more than one taxon together in the same unclassified "bin". They are presented here just for the purpose of showing the raw results from the analysis pipeline.

Supplementary Table 2

Bacterial Genus	Metabolite Peak ID	Metabolite MSI 3 ID	Class	p2.5	Mean	p97.5
Rhodospirillaceae_(unclassified _genus)	X490	PA(O-20:0/13:0)	Glycerophospholipid	-0.679120143289078	**-0.521321846091987**	-0.329428775902636
Rhodospirillaceae_(unclassified _genus)	X458	20:2-Glc-Campesterol	Sterol lipid	-0.650369650206592	**-0.473331569074881**	-0.266645173134682
Megasphaera	X7183	Beta Alanine	Carboxylic acid	-0.649653311587389	**-0.469037952275615**	-0.249846313984503
Rhodospirillaceae_(unclassified _genus)	X331	Cholesterol sulfate	Steroid and derivaties	-0.644896333372774	**-0.468591234473163**	-0.269457703318087
Rhodospirillaceae_(unclassified _genus)	X418	Sorbitan stearate	Sorbitol derivative	-0.641777000809738	**-0.467513104508021**	-0.264742498780816
Ruminococcus2	X7285	16alpha-hydroxy-estrone	Steroid and derivaties	-0.638066684951759	**-0.467295124713299**	-0.276775275924102
Rhodospirillaceae_(unclassified _genus)	X70	(3S,5R,6S,7E,9x)-7-Megastigmene-3,6,9-triol 9-glucoside	Fatty acyl glycoside	-0.626542825537098	**-0.454671954200975**	-0.258293309239509
Rhodospirillaceae_(unclassified _genus)	X313	Iodovulone I	Chemical	-0.622464822131849	**-0.435875152609181**	-0.230646228350224
Megasphaera	X7195	Tridecane	Saturated hydrocarbon	-0.617751405367128	**-0.426024257015954**	-0.207065143643662
Megasphaera	X7264	N-acetyl-cysteine	Drug or drug metabolite	-0.615637991911478	**-0.422580887485948**	-0.196530058381189
Rhodospirillaceae_(unclassified _genus)	X5	Iodovulone I	Chemical	-0.590143590345582	**-0.416348444915009**	-0.204096199578182
Asaccharobacter	X115	3-hydroxybutyrylcarnitine	Acylcarnitines	-0.596756314110753	**-0.415665011470388**	-0.201423242107011
Megasphaera	X7175	Dodecane	Alkane	-0.60893308996141	**-0.412642019157781**	-0.186330002604256
Asaccharobacter	X276	SM(d18:0/24:0)	Sphingolipid	-0.598727116787475	**-0.410632321835523**	-0.200611657726504
Asaccharobacter	X313	Iodovulone I	Chemical	-0.594164611537273	**-0.405383817289153**	-0.189543562901321
Alphaproteobacteria_(unclassified_order)	X263	Epigallocatechin 3-O-caffeate	Epigallocatechins	-0.596037026414238	**-0.403182935039362**	-0.181371036561443
Rhodospirillaceae_(unclassified _genus)	X276	SM(d18:0/24:0)	Sphingolipid	-0.576461570078292	**-0.393987994517586**	-0.184678772325972
Megasphaera	X7191	N-formyl-methionine	Carboxylic acid and derivatives	-0.641656427116156	**-0.393679580794709**	-0.135390532693707
Asaccharobacter	X429	PC(16:0/18:1(6Z))	Glycerophospholipid	-0.587230470957046	**-0.392930866419923**	-0.171893191703785
Butyrivibrio	X7280	Heptadecane	Alkane	-0.578008560702705	**-0.387208245933954**	-0.164608787658533
Pasteurellaceae_(unclassified_genus)	X7285	16alpha-hydroxy-estrone	Steroid and derivaties	-0.563880458639557	**-0.381534202586008**	-0.164572802545422
Rhodospirillaceae_(unclassified _genus)	X420	PC(16:1(9Z)/0:0)	Glycerophospholipid	-0.574148637781262	**-0.378841081665662**	-0.163058133824681
Rhodospirillaceae_(unclassified _genus)	X500	Butyrylcarnitine	Fatty acyls	-0.571940819933159	**-0.378161526679644**	-0.161043733382863
Acidaminococcaceae_(unclassified_genus)	X7281	Benzaldehyde	Benzoids	-0.565268499119518	**-0.374593293729041**	-0.161751948370262
Rikenellaceae_(unclassified_genus)	X6277	Veranisatin C	Prenol lipids	-0.561130153519398	**-0.374148920001944**	-0.15463293929418
Ruminococcus	X6174	N2,N2-Dimethylguanosine	Purine nucleosides	-0.564878466851899	**-0.373809448944771**	-0.1620937774711
Rhodospirillaceae_(unclassified _genus)	X327	Anandamide (20:5, n-3)	Fatty acid amide	-0.575284463215288	**-0.372133944176319**	-0.158726156774862
Desulfovibrionales_(unclassified _family)	X32	Jolkinol B	Chemical	-0.582362776483108	**-0.366368285220238**	-0.0998514030441841
Rhodospirillaceae_(unclassified _genus)	X32	Jolkinol B	Chemical	-0.563565637272992	**-0.366126986963159**	-0.146449281629806
Turicibacter	X24	Cycloheterophyllin	Pyranoflavonoids	-0.581811127154801	**-0.36358219105731**	-0.123371274815158
Turicibacter	X13	Leukotriene D5	O+D311rganooxygen compounds	-0.559342453800966	**-0.363439709679447**	-0.136134061857679
Megasphaera	X7279	Proline	Carboxylic acid and derivatives	-0.574233983779206	**-0.363162940248775**	-0.131372096712824
Coriobacteriaceae_(unclassified _genus)	X311	Sphingofungin A	Antifungal	-0.54936779648548	**-0.362695664568241**	-0.150729165650657
Asaccharobacter	X70	(3S,5R,6S,7E,9x)-7-Megastigmene-3,6,9-triol 9-glucoside	Fatty acyl glycoside	-0.561910108989562	**-0.362398648867187**	-0.140790046214654
Phascolarctobacterium	X313	Iodovulone I	Chemical	-0.557023270864119	**-0.359866449929682**	-0.13297599044737
Catenibacterium	X7175	Dodecane	Alkane	-0.561847170267903	**-0.353872554722291**	-0.111980880972984

Rhodospirillaceae_(unclassified_genus)	X144	1alpha,24,25,28-tetrahydroxyergocalciferol	Vitamin D metabolite	-0.55099839343902	**-0.351940915583564**	-0.125963968097654
Turicibacter	X320	11-cis-Dehydroretinal	Derivative of Vitamin A2	-0.550455941529851	**-0.350721214666453**	-0.117722854387576
Megasphaera	X7160	Dodecane	Alkane	-0.562408183585084	**-0.348198351511365**	-0.110175825897467
Rhodospirillaceae_(unclassified_genus)	X287	Fuconic acid	Chemical	-0.54431087788658	**-0.347215997262819**	-0.118939189809549
Asaccharobacter	X130	7,3'-Dihydroxy-4'-methoxy-8-methylflavan	Flavonoids	-0.543825776286605	**-0.346703730970603**	-0.118845091560781
Clostridia_(unclassified_order)	X6808	OKHdiA-PS	Chemical	-0.545368863043117	**-0.345635321570586**	-0.119121465402648
Rhodospirillaceae_(unclassified_genus)	X502	PE(P-18:0/20:5(5Z,8Z,11Z,14Z,17Z))	Glycerophospholipid	-0.54536999178665	**-0.342962157829289**	-0.126168641703009
Acidaminococcus	X7232	Norvaline	Amino acid	-0.538435993603962	**-0.341399493824132**	-0.133879020264168
Dialister	X75	Stearoyl tyrosine	Tyrosine and derivatives	-0.529377662744139	**-0.340808970460395**	-0.128895263896772
Asaccharobacter	X443	GlcCer(d18:1(8E)/21:0(2OH[R]))	Sphingolipid	-0.539304602134743	**-0.340005941389037**	-0.108564842635302
Asteroleplasma	X7250	Urea	Organic acids and derivatives	-0.549275728766687	**-0.339942227244316**	-0.101062234641267
Holdemanella	X9	OHOHA-PS	Chemical	-0.543775645541524	**-0.339536462472111**	-0.0964931531774862
Phascolarctobacterium	X443	GlcCer(d18:1(8E)/21:0(2OH[R]))	Sphingolipid	-0.529657430554356	**-0.338726646379111**	-0.125295808211819
Rhodospirillaceae_(unclassified_genus)	X399	PE(O-20:0/22:6(4Z,7Z,10Z,13Z,16Z,19Z))	Glycerophospholipid	-0.545770507340746	**-0.33800507697358**	-0.10876844664941
Asteroleplasma	X7183	Beta Alanine	Carboxylic acid	-0.539282977244087	**-0.337689226040773**	-0.109060324512156
Howardella	X511	PC(18:3(9Z,12Z,15Z)/0:0)	Glycerophospholipid	-0.540349985804896	**-0.337621683119202**	-0.110106530083215
Collinsella	X7246	4-hydroxy-, trans-proline	Carboxylic acid and derivatives	-0.534139481705561	**-0.337499943109439**	-0.122980332533725
Asaccharobacter	X331	Cholesterol sulfate	Steroid and derivaties	-0.541760313918567	**-0.336990922904438**	-0.106081042617492
Asaccharobacter	X420	PC(16:1(9Z)/0:0)	Glycerophospholipid	-0.540612740956616	**-0.335911198875649**	-0.112386587093049
Firmicutes_(unclassified_class)	X6176	3,3-Dibromo-2-n-hexylacrylic acid	Fatty acyls	-0.535168162116922	**-0.335046729935324**	-0.126834548420074
Eubacterium	X7285	16alpha-hydroxy-estrone	Steroid and derivaties	-0.527935988638004	**-0.334338463697501**	-0.0910445778452158
Rhodospirillaceae_(unclassified_genus)	X406	SM(d16:1/22:0)	Sphingolipid	-0.540261353263354	**-0.333222058016197**	-0.107599281846838
Porphyromonas	X170	PA(P-18:0/17:2(9Z,12Z))	Glycerophospholipid	-0.556235849576343	**-0.332430207268524**	-0.0832481014025988
Odoribacter	X24	Cycloheterophyllin	Pyranoflavonoids	-0.527142362194719	**-0.330936137984471**	-0.113520380242465
Rhodospirillaceae_(unclassified_genus)	X497	SM(d17:1/24:1)	Sphingolipid	-0.539592225097223	**-0.330592249560755**	-0.0996670813877792
Clostridia_(unclassified_order)	X32	Jolkinol B	Chemical	-0.535115133472631	**-0.329468764630719**	-0.10763597737408
Catenibacterium	X7195	Tridecane	Saturated hydrocarbon	-0.547211760894441	**-0.329355283695667**	-0.0871688968967284
Dialister	X499	3-Deoxyvitamin D3	Sterol lipid	-0.52739304424054	**-0.329347753819252**	-0.111722810489366
Parasutterella	X368	Deca-4,6,8-triyne-1,1,2,3-tetraol	Artificial chemical	-0.520769347472861	**-0.328759142936378**	-0.112818413797355
Clostridium_XVIII	X418	Sorbitan stearate	Sorbitol derivative	-0.520193688104859	**-0.32818029897233**	-0.114265105767602
Rhodospirillaceae_(unclassified_genus)	X362	PC(22:4(7Z,10Z,13Z,16Z)/0:0)	Glycerophospholipid	-0.533302956280858	**-0.327699723542642**	-0.0974493389930356
Eisenbergiella	X6288	Sphingosine-1-phosphate	Phosphosphingolipids	-0.520067044747608	**-0.327235109329465**	-0.107167517651896
Erysipelotrichaceae_(unclassified_genus)	X7285	16alpha-hydroxy-estrone	Steroid and derivaties	-0.522033694880292	**-0.325081820853406**	-0.109567687706961
Veillonella	X7285	16alpha-hydroxy-estrone	Steroid and derivaties	-0.512379342202291	**-0.322361639506064**	-0.109784685890788
Escherichia/Shigella	X7285	16alpha-hydroxy-estrone	Steroid and derivaties	-0.519380921252199	**-0.321519780036984**	-0.0894576001440653
Flavonifractor	X7285	16alpha-hydroxy-estrone	Steroid and derivaties	-0.51724631469304	**-0.320405608011138**	-0.115236001739349
Romboutsia	X7206	N-formyl-methionine	Carboxylic acid and derivatives	-0.513260893982776	**-0.320293434037722**	-0.0901323865863124
Paraprevotella	X9	OHOHA-PS	Chemical	-0.521498394895086	**-0.319945140965186**	-0.0940496110418401
Catenibacterium	X7279	Proline	Carboxylic acid and derivatives	-0.533508889125571	**-0.319401817040117**	-0.0650965812757318

Megasphaera	X7186	5-methyl-hydantoin	Chemical	-0.539855177861322	**-0.317921150309555**	-0.0796492388300585
Catenibacterium	X7278	Tetradecane	Saturated hydrocarbon	-0.544840705408374	**-0.317797511182356**	-0.0521748198674437
Megasphaera	X7205	Serine	Amino acid	-0.533555267008254	**-0.317618076312541**	-0.0615538942439306
Romboutsia	X7145	PI-Cer(t20:0/22:0(2OH))	Glycerophospholipid	-0.514999328705362	**-0.317049188971376**	-0.100415330126664
Howardella	X349	GalCer(d18:1/23:0);GlcCer(d18:1/23:0)	Sphingolipid	-0.532147106771172	**-0.315307654946345**	-0.0855138087779663
Phascolarctobacterium	X144	1alpha,24,25,28-tetrahydroxyergocalciferol	Vitamin D metabolite	-0.501466130384715	**-0.314474857464416**	-0.0908049857243348
Rhodospirillaceae_(unclassified_genus)	X6176	3,3-Dibromo-2-n-hexylacrylic acid	Fatty acyls	-0.53247716360209	**-0.313608333884066**	-0.0779768151199533
Rhodospirillaceae_(unclassified_genus)	X498	SM(d18:2/21:0)	Sphingolipid	-0.52346153172896	**-0.313596667406169**	-0.0871582465967042
Phascolarctobacterium	X331	Cholesterol sulfate	Steroid and derivaties	-0.50617718268849	**-0.312554619346302**	-0.0799587493076379
Megasphaera	X7241	3,4-dimethyl-aniline	Arylamine	-0.540456522463158	**-0.312414235890742**	-0.0320906056124488
Dialister	X36	PG(16:1(9Z)/22:4(7Z,10Z,13Z,16Z))	Glycerophospholipid	-0.512469307030068	**-0.311990433171758**	-0.0963380766503928
Alphaproteobacteria_(unclassified_order)	X474	Acevaltrate	Carboxylic acid	-0.52594800292966	**-0.311561483360308**	-0.0630408729538175
Barnesiella	X130	7,3'-Dihydroxy-4'-methoxy-8-methylflavan	Flavonoids	-0.508808275718308	**-0.311220347209953**	-0.0896174077892957
**Bifidobacterium**	X7163	2-amino-2-deoxy-glucose	Hexoses	-0.504336709004767	**-0.310931208985688**	-0.0854462694046852
Faecalicoccus	X443	GlcCer(d18:1(8E)/21:0(2OH[R]))	Sphingolipid	-0.518163484680327	**-0.309797886855962**	-0.0862609236012807
Flavonifractor	X6877	5-Methyltetrahydropteroyltri-L-glutamate	Steroid and derivaties	-0.501774342122627	**-0.309521173947209**	-0.0966462588864618
Anaerotruncus	X7246	4-hydroxy-, trans-proline	Carboxylic acid and derivatives	-0.499571036385806	**-0.309496110857305**	-0.0837871312687374
Catenibacterium	X7183	Beta Alanine	Carboxylic acid	-0.527709543770616	**-0.308538738781953**	-0.0735776954644683
Turicibacter	X439	PI(16:0/20:1(11Z))	Glycerophospholipid	-0.519463504551724	**-0.308503389691947**	-0.0629808727895828
Megasphaera	X7287	Tartronic acid	Dicarboxylic acid	-0.523266328431971	**-0.307961995698902**	-0.0678637379727717
Gemmiger	X75	Stearoyl tyrosine	Tyrosine and derivatives	-0.506411687870344	**-0.307495926471119**	-0.084135719142613
Senegalimassilia	X7003	MGDG(20:5(5Z,8Z,11Z,14Z,17Z)/18:3(9Z,12Z,15Z))	Glycerolipid	-0.535315211519146	**-0.307474741698095**	-0.0694267939984785
Eisenbergiella	X7285	16alpha-hydroxy-estrone	Steroid and derivaties	-0.508690438761814	**-0.307345450098921**	-0.087139919272076
Clostridium_XlVb	X7207	Maltotriose	Oligosaccharides	-0.512034379514076	**-0.307094898283421**	-0.082804702953984
Porphyromonas	X348	Fuconic acid	Chemical	-0.518267647873876	**-0.30614476961838**	-0.0623471406854592
Turicibacter	X407	PE(20:2(11Z,14Z)/0:0)	Glycerophospholipid	-0.523623312206428	**-0.305957772508099**	-0.0645161461347392
Butyrivibrio	X6984	PE(20:4(8Z,11Z,14Z,17Z)/20:4(8Z,11Z,14Z,17Z))	Glycerophospholipid	-0.523301586439835	**-0.305600419545478**	-0.0685254048452729
Succiniclasticum	X7280	Heptadecane	Alkane	-0.521544824561509	**-0.304293119739756**	-0.0724873853261455
Phascolarctobacterium	X6092	3,4-dimethyl-5-carboxyethyl-2-furanpentanoic acid	Furanoic fatty acids	-0.496840375552616	**-0.303999214956524**	-0.0744181037880483
Peptococcus	X7176	3-Methyl-2-oxopentanoic-acid	Neurotoxin	-0.506094784338612	**-0.300750128596512**	-0.079313878150706
Turicibacter	X337	C17 sphingosine-1-phosphocholine	Sphingolipid	-0.517482743948174	**-0.300497482991974**	-0.0532490890588934
Acidaminococcus	X6786	OHOHA-PS	Chemical	0.0599494082294354	**0.300650464950199**	0.524259217032965
Puniceicoccaceae_(unclassified_genus)	X7163	2-amino-2-deoxy-glucose	Hexoses	0.0401795065896619	**0.300744665944609**	0.543554302535459
Clostridia_(unclassified_order)	X7232	Norvaline	Amino acid	0.0403953201645206	**0.300795829803444**	0.561091601185555
Turicibacter	X313	Iodovulone I	Chemical	0.0676290765148755	**0.302166065032344**	0.518263074376637
Rhodospirillaceae_(unclassified_genus)	X439	PI(16:0/20:1(11Z))	Glycerophospholipid	0.0695065261709846	**0.302179012032754**	0.513255287359888
Desulfovibrio	X13	Leukotriene D5	Organooxygen compounds	0.0637944767430523	**0.302188944828459**	0.517729926840126
Howardella	X502	PE(P-18:0/20:5(5Z,8Z,11Z,14Z,17Z))	Glycerophospholipid	0.0667775175779412	**0.302565756946585**	0.522749056274586
Mollicutes_(unclassified_order)	X429	PC(16:0/18:1(6Z))	Glycerophospholipid	0.0397664767733599	**0.302680577143249**	0.538193337401686

Pseudoflavonifractor	X7175	Alkane	Alkane	0.0374963259466729	**0.303531187353179**	0.549481208263126
Rikenellaceae_(unclassified_genus)	X7164	2-amino-2-deoxy-glucose	Glucose derivative	0.0488145344024966	**0.305106514117545**	0.526092186699854
Clostridia_(unclassified_order)	X348	Fuconic acid	Chemical	0.0822128144031839	**0.305528361735329**	0.510499609735394
Mollicutes_(unclassified_order)	X130	7,3'-Dihydroxy-4'-methoxy-8-methylflavan	Flavonoids	0.0398654363658816	**0.306153867199384**	0.537187476246348
Burkholderiales_(unclassified_family)	X276	SM(d18:0/24:0)	Sphingolipid	0.0664106107481085	**0.307256809206456**	0.515295199734897
Desulfovibrio	X177	Glutarylcarnitine	Acylcarnitines	0.0781408902289617	**0.307950997212865**	0.527856410988292
Howardella	X115	3-hydroxybutyrylcarnitine	Acylcarnitines	0.0702303568341303	**0.30803915047287**	0.522570238499886
Butyricimonas	X463	PC(P-16:0/20:3(8Z,11Z,14Z))	Glycerophospholipid	0.0836093785312831	**0.308775637988643**	0.512441084072855
Sutterella	X264	6-Keto-decanoylcarnitine	Fatty acyls	0.0731793864780109	**0.308789827030504**	0.5128195696662
Sutterella	X6174	N2,N2-Dimethylguanosine	Purine nucleosides	0.0932757624881701	**0.308799425307756**	0.506712062764001
Bacteria_(unclassified_phylum)	X7232	Norvaline	Amino acid	0.0868982606946315	**0.308892295821145**	0.503454679188788
Proteobacteria_(unclassified_class)	X485	SM(d18:1/21:0)	Sphingolipid	0.0592577850498874	**0.310775171633633**	0.522202991063303
Faecalicoccus	X7191	N-formyl-methionine	Carboxylic acid and derivatives	0.0795145050622052	**0.313482097802345**	0.519719955025861
Holdemanella	X327	Anandamide (20:5, n-3)	Fatty acid amide	0.0686865209198179	**0.313785090457429**	0.534550498107193
Bacteroidetes_(unclassified_class)	X191	Sphingosine-1-phosphate	Phosphosphingolipids	0.0767876751373597	**0.313998108957262**	0.518979175717173
Anaerostipes	X7244	Citrulline	Carboxylic acid and derivatives	0.0854042333292754	**0.315087111523073**	0.516260281926663
Dorea	X6808	OKHdiA-PS	Chemical	0.0861615776901488	**0.31542098536167**	0.51101516515329
Rhodospirillaceae_(unclassified_genus)	X243	PS(20:3(8Z,11Z,14Z)/0:0)	Glycerophospholipid	0.0658407569048098	**0.315471796803388**	0.523521323980807
Mollicutes_(unclassified_order)	X70	(3S,5R,6S,7E,9x)-7-Megastigmene-3,6,9-triol 9-glucoside	Fatty acyl glycoside	0.0572959292195704	**0.316524246282256**	0.546397290499767
Clostridium_XVIII	X7276	2-amino-2-deoxy-, D-Galactose	Galactose derivative	0.0805093048330435	**0.318234342449635**	0.546374636661269
Clostridia_(unclassified_order)	X7191	N-formyl-methionine	Carboxylic acid and derivatives	0.0871979456818575	**0.318530204505525**	0.522145867550151
Asteroleplasma	X511	PC(18:3(9Z,12Z,15Z)/0:0)	Glycerophospholipid	0.0673058415470098	**0.318846025896461**	0.527554303416781
Turicibacter	X500	Butyrylcarnitine	Fatty acyls	0.0845044854401082	**0.319186833461802**	0.532909982253882
Mollicutes_(unclassified_order)	X348	Fuconic acid	Chemical	0.0640661800967755	**0.319192959987919**	0.545377187073613
Pseudoflavonifractor	X7160	Dodecane	Alkane	0.0764028892861346	**0.319917411468846**	0.530894403793541
Rhodospirillaceae_(unclassified_genus)	X511	PC(18:3(9Z,12Z,15Z)/0:0)	Glycerophospholipid	0.0858202421499787	**0.321191643527858**	0.526531928613218
Holdemanella	X443	GlcCer(d18:1(8E)/21:0(2OH[R]))	Sphingolipid	0.0743751612882899	**0.321621751710061**	0.535918749698891
Phascolarctobacterium	X407	PE(20:2(11Z,14Z)/0:0)	Glycerophospholipid	0.103605269532063	**0.321696336863525**	0.507590339648016
Holdemanella	X490	PA(O-20:0/13:0)	Glycerophospholipid	0.0818724851283757	**0.322398363213787**	0.544374573176528
Mollicutes_(unclassified_order)	X276	SM(d18:0/24:0)	Sphingolipid	0.0724792020968001	**0.323237368570567**	0.55769661798844
Intestinimonas	X6065	Palmitelaidic acid	Fatty acyls	0.117836391153954	**0.323675047369813**	0.518266650224458
Firmicutes_(unclassified_class)	X7164	2-amino-2-deoxy-glucose	Glucose derivative	0.113474390324083	**0.324058004351464**	0.520658187243102
Howardella	X490	PA(O-20:0/13:0)	Glycerophospholipid	0.0905226776618946	**0.324950306960029**	0.536765498014835
Klebsiella	X6610	2-methylbacteriohopane-32,33,34,35-tetrol	Prenol lipids	0.0857127569263566	**0.325753666212876**	0.538621532029568
Rhodospirillaceae_(unclassified_genus)	X7287	Tartronic acid	Dicarboxylic acid	0.0774706903408894	**0.325759893840917**	0.543239838394745
Desulfovibrionaceae_(unclassified_genus)	X7160	Dodecane	Alkane	0.100836202619415	**0.326179901867341**	0.538177067814268
Deltaproteobacteria_(unclassified_order)	X463	PC(P-16:0/20:3(8Z,11Z,14Z))	Glycerophospholipid	0.0937420420536071	**0.326226888033477**	0.537677512643142
Holdemanella	X130	7,3'-Dihydroxy-4'-methoxy-8-methylflavan	Flavonoids	0.0881001240685059	**0.326848594496742**	0.548443266299542
Firmicutes_(unclassified_class)	X7160	Dodecane	Alkane	0.108985676397234	**0.327778839952697**	0.518886526084322

Clostridium_sensu_stricto	X7276	2-amino-2-deoxy-, D-Galactose	Galactose derivative	0.105984355020647	**0.328435055005004**	0.534125403517243
Klebsiella	X6237	Unknown	Unknown	0.0994605114827311	**0.329660207102423**	0.54358742142114
Lachnospira	X6877	5-Methyltetrahydropteroyltri-L-glutamate	Steroid and derivaties	0.109384772748499	**0.33022579082687**	0.523814209407503
Asteroleplasma	X363	3-carboxy-4-methyl-5-pentyl-2-furanpropanoic acid	F+D151uranoic fatty acids	0.0856218432655642	**0.330570424092658**	0.546165729303708
Dialister	X348	Fuconic acid	Chemical	0.117647325697005	**0.33191782231121**	0.522858847556443
Desulfovibrionales_(unclassified_family)	X7191	N-formyl-methionine	Carboxylic acid and derivatives	0.0693154718363847	**0.333516968350083**	0.566402928092143
Rikenellaceae_(unclassified_genus)	X7175	Alkane	Alkane	0.0839536495584148	**0.334229029474645**	0.565533082138604
Howardella	X130	7,3'-Dihydroxy-4'-methoxy-8-methylflavan	Flavonoids	0.0907478069254011	**0.334382103696908**	0.552261430260681
Holdemanella	X458	20:2-Glc-Campesterol	Sterol lipid	0.0970048079343878	**0.334736523677968**	0.550175856540918
Mollicutes_(unclassified_order)	X313	Iodovulone I	Chemical	0.0841827375215808	**0.335965181412452**	0.557229854031709
Victivallis	X7164	2-amino-2-deoxy-glucose	Glucose derivative	0.11012430304715	**0.338657209153354**	0.531840507307177
Turicibacter	X362	PC(22:4(7Z,10Z,13Z,16Z)/0:0)	Glycerophospholipid	0.105741870772995	**0.338847304938495**	0.545182240072361
Pseudoflavonifractor	X7287	Tartronic acid	Dicarboxylic acid	0.0924781824660241	**0.342552662046565**	0.573685936997778
Turicibacter	X406	SM(d16:1/22:0)	Sphingolipid	0.108348317904275	**0.342820543569845**	0.546392692662474
Holdemanella	X276	SM(d18:0/24:0)	Sphingolipid	0.0989330402773034	**0.343072820173102**	0.565381565256944
Collinsella	X6786	OHOHA-PS	Chemical	0.130323346532524	**0.344019296128269**	0.54707219211187
Klebsiella	X6860	PE(16:0/P-18:1(11Z))	Glycerophospholipid	0.118060018780107	**0.345330346649566**	0.541767403243386
Turicibacter	X399	PE(O-20:0/22:6(4Z,7Z,10Z,13Z,16Z,19Z))	Glycerophospholipid	0.10869422293362	**0.345596873342676**	0.554643619613606
Holdemanella	X331	Cholesterol sulfate	Steroid and derivaties	0.111572135635709	**0.345746310328568**	0.555200823519786
Howardella	X458	20:2-Glc-Campesterol	Sterol lipid	0.111430912459642	**0.345847781216353**	0.549718449639825
Sutterella	X263	Epigallocatechin 3-O-caffeate	Epigallocatechins	0.125574171304026	**0.347616627958984**	0.550783758082746
Mollicutes_(unclassified_order)	X115	3-hydroxybutyrylcarnitine	Acylcarnitines	0.104547939160705	**0.350453111391825**	0.575402647166465
Mollicutes_(unclassified_order)	X443	GlcCer(d18:1(8E)/21:0(2OH[R]))	Sphingolipid	0.100204653488125	**0.353607142676098**	0.564171465965258
Desulfovibrio	X414	PA(O-16:0/21:0)	Glycerophospholipid	0.12806246548032	**0.354937821028586**	0.560234999234703
Turicibacter	X420	PC(16:1(9Z)/0:0)	Glycerophospholipid	0.118438635437031	**0.356662445806884**	0.56949143325013
Escherichia/Shigella	X485	SM(d18:1/21:0)	Sphingolipid	0.143628005667532	**0.356745968495558**	0.542556488138927
Turicibacter	X144	1alpha,24,25,28-tetrahydroxyergocalciferol	Vitamin D metabolite	0.125391719906216	**0.356901062014273**	0.570807547785395
Rikenellaceae_(unclassified_genus)	X7195	Saturated hydrocarbon	Alkane	0.101574143489115	**0.357009886502931**	0.58579631982132
Clostridium_XVIII	X7186	5-methyl-hydantoin	Chemical	0.136667375081109	**0.357721304178808**	0.558031998256618
Streptococcus	X6984	PE(20:4(8Z,11Z,14Z,17Z)/20:4(8Z,11Z,14Z,17Z))	Glycerophospholipid	0.143276391353653	**0.358968774012197**	0.54305386235902
Howardella	X331	Cholesterol sulfate	Steroid and derivaties	0.139174106785519	**0.367182262335715**	0.573077426104364
Rhodospirillaceae_(unclassified_genus)	X7164	2-amino-2-deoxy-glucose	Glucose derivative	0.14732391800147	**0.367185241952125**	0.571116740323407
Bacteria_(unclassified_phylum)	X7281	Benzaldehyde	Benzoids	0.158936981083975	**0.367655328945032**	0.554336245798987
Howardella	X327	Anandamide (20:5, n-3)	Fatty acid amide	0.136454079129513	**0.367773228240198**	0.573364763350332
Holdemanella	X313	Iodovulone I	Chemical	0.143126114299911	**0.376425075515487**	0.57787928439828
Holdemanella	X70	(3S,5R,6S,7E,9x)-7-Megastigmene-3,6,9-triol 9-glucoside	Fatty acyl glycoside	0.140358200904432	**0.379827403198058**	0.581507704919094
Holdemanella	X418	Sorbitan stearate	Sorbitol derivative	0.158552109892461	**0.384264733851717**	0.585556731595976
Desulfovibrio	X368	Deca-4,6,8-triyne-1,1,2,3-tetraol	Artificial chemical	0.155504692343396	**0.386257117658749**	0.580544573447888
Howardella	X70	(3S,5R,6S,7E,9x)-7-Megastigmene-3,6,9-triol 9-glucoside	Fatty acyl glycoside	0.159793259973888	**0.387098066105368**	0.596906963336397

Howardella	X276	SM(d18:0/24:0)	Sphingolipid	0.156118044522978	**0.391431545937212**	0.585962618135185
Coriobacteriaceae_(unclassified _genus)	X13	Leukotriene D5	Organooxygen compounds	0.182196794710891	**0.398885947863281**	0.582653159202683
Barnesiella	X499	3-Deoxyvitamin D3	Sterol lipid	0.185857859420496	**0.401772289331714**	0.584295082763588
Pseudoflavonifractor	X7191	N-formyl-methionine	Carboxylic acid and derivatives	0.149879075801403	**0.402187219359384**	0.609432172984734
Howardella	X313	Iodovulone I	Chemical	0.18541193011122	**0.406161015884899**	0.600779634921908
Rhodospirillaceae_(unclassified _genus)	X7160	Dodecane	Alkane	0.194757618496707	**0.408594358107515**	0.597842361744379
Howardella	X418	Sorbitan stearate	Sorbitol derivative	0.190605375891309	**0.411157940189298**	0.605426271024193
Desulfovibrio	X170	PA(P-18:0/17:2(9Z,12Z))	Glycerophospholipid	0.19044484526383	**0.412476195331561**	0.596591358893752
Rhodospirillaceae_(unclassified _genus)	X7191	N-formyl-methionine	Carboxylic acid and derivatives	0.20812932071626	**0.424222278705709**	0.605586654606276
Desulfovibrio	X47	PE(18:4(6Z,9Z,12Z,15Z)/15:1(9Z))	Glycerophospholipid	0.206734979074421	**0.426877580188305**	0.6138154236854

NOTE: Unclassified taxa may contain more than one taxon together in the same unclassified "bin". They are presented here just for the purpose of showing the raw results from the analysis pipeline.

Supplementary Table 3

Family	Metabolite Peak ID	Metabolite MSI 3 ID	p2.5	Correlation	p97.5
Mollicutes_(unclassified_order)	X170	PA(P-18:0/17:2(9Z,12Z))	-0.618286229801283	-0.395445228880232	-0.126202740109402
Anaeroplasmataceae	X276	SM(d18:0/24:0)	-0.579330401449037	-0.390454747279393	-0.17766180536865
Mollicutes_(unclassified_order)	X320	11-cis-Dehydroretinal;all-trans-Dehydroretinal	-0.584581471343472	-0.377933173060803	-0.132008774724165
Mollicutes_(unclassified_order)	X337	C17 sphingosine-1-phosphocholine	-0.591402469405223	-0.377362322857814	-0.136420822441862
Anaeroplasmataceae	X70	(3S,5R,6S,7E,9x)-7-Megastigmene-3,6,9-triol 9-glucoside	-0.571129739279383	-0.373018699222295	-0.151282199595801
Burkholderiales_(unclassified_family)	X6288	Sphingosine-1-phosphate;Sphingosine 1-phosphate	-0.555003873803152	-0.366153837150236	-0.155268228552797
Mollicutes_(unclassified_order)	X13	Leukotriene D5	-0.567563351314284	-0.364516731153215	-0.118597702196464
Verrucomicrobiaceae	X7038	PE(20:2(11Z,14Z)/22:5(4Z,7Z,10Z,13Z,16Z))	-0.543044891225729	-0.357886548343295	-0.149589650878171
Desulfovibrionales_(unclassified_family)	X459	PC(P-18:0/20:5(5Z,8Z,11Z,14Z,17Z))	-0.560952942620982	-0.351882328142162	-0.119219718704237
Victivallaceae	X7206	Methionine, N-formyl-	-0.539851073364413	-0.349726446703867	-0.133405658167099
Burkholderiales_(unclassified_family)	X7263	Glycine, 2-phenyl-	-0.547716542056285	-0.348620389360129	-0.132979220477838
Rhodospirillales_(unclassified_family)	X361	3,4-dimethyl-5-carboxyethyl-2-furanhexanoic acid	-0.545767338719944	-0.345624478486515	-0.126727706087735
Anaeroplasmataceae	X6319	Galbanic acid	-0.539653520187514	-0.344961195966059	-0.112982906125725
Anaeroplasmataceae	X331	cholesterol sulfate	-0.547111083568355	-0.343067890591901	-0.10977756931713
Anaeroplasmataceae	X144	1alpha,24,25,28-tetrahydroxyergocalciferol	-0.542229712481619	-0.341768766433096	-0.105477783292878
Desulfovibrionales_(unclassified_family)	X518	PC(18:4(6Z,9Z,12Z,15Z)/18:1(11Z))	-0.550326803903	-0.340035358213387	-0.100926491861573
Mollicutes_(unclassified_order)	X295	Glycoursodeoxycholic acid	-0.552944146960957	-0.338163228867074	-0.084854197919047
Bacteroidetes_(unclassified_class)	X327	Anandamide (20:5, n-3)	-0.543964424581174	-0.335286422049681	-0.105754391581413
Victivallaceae	X430	PC(P-20:0/18:3(6Z,9Z,12Z))	-0.529492594991057	-0.33520309871688	-0.11624913897319
Synergistaceae	X348	Fuconic acid	-0.549125214871763	-0.334752847408137	-0.09364833327076
Clostridia_(unclassified_order)	X429	PC(16:0/18:1(6Z));PC(16:0/18:1(6E))	-0.532317492539323	-0.334156091015832	-0.129017736485216
Anaeroplasmataceae	X420	PC(16:1(9Z)/0:0);PC(16:1(9E)/0:0)	-0.536222648133319	-0.332531405154864	-0.106658176089306
Clostridia_(unclassified_order)	X276	SM(d18:0/24:0)	-0.521419061533868	-0.330256270234448	-0.112385862985559
Anaeroplasmataceae	X115	Hydroxybutyrylcarnitine;3-hydroxybutyrylcarnitine	-0.533424078078089	-0.329217664271316	-0.104368676669298
Mollicutes_(unclassified_order)	X439	PI(16:0/20:1(11Z))	-0.558077643722219	-0.328929446781473	-0.076935687033778
Anaeroplasmataceae	X443	GlcCer(d18:1(8Z)/21:0(2OH[R]));GlcCer(d18:1(8E)/21:0(2OH[R]))	-0.527162729435238	-0.328863689045159	-0.092065858819192
Mollicutes_(unclassified_order)	X67	4-O-alpha-Cadinylangolensin	-0.539043166263066	-0.325138670505131	-0.07419512525394
Anaeroplasmataceae	X362	PC(22:4(7Z,10Z,13Z,16Z)/0:0)	-0.528421081670498	-0.320063097448397	-0.082518076142815
Clostridiaceae_1	X7236	Proline, 4-hydroxy-, trans-	-0.513396467002744	-0.318777055072178	-0.103859315083166
Bacteria_(unclassified_phylum)	X6277	Veranisatin C	-0.509927877452388	-0.31841610475978	-0.104807743582324
Clostridia_(unclassified_order)	X130	7,3'-Dihydroxy-4'-methoxy-8-methylflavan	-0.513262544990155	-0.31659614774081	-0.097303119547862
Alphaproteobacteria_(unclassified_order)	X511	PC(18:3(9Z,12Z,15Z)/0:0)	-0.53743151525348	-0.315358682582585	-0.070142118857877
Bacteroidetes_(unclassified_class)	X518	PC(18:4(6Z,9Z,12Z,15Z)/18:1(11Z))	-0.515020199432491	-0.315125848647006	-0.095269183265422
Anaeroplasmataceae	X327	Anandamide (20:5, n-3)	-0.528483727975029	-0.314879090939818	-0.071469877632687
Anaeroplasmataceae	X313	iodovulone I	-0.523426777371549	-0.313590779813789	-0.087559848129202

Anaeroplasmataceae	X130	7,3'-Dihydroxy-4'-methoxy-8-methylflavan	-0.513131411514349	-0.310866672079885	-0.076437652143976
Rhodospirillales_(unclassified_family)	X7170	n-tricosane	-0.50788445287186	-0.310368482813186	-0.091833410915208
Clostridiales_Incertae_Sedis_XI	X7186	Hydantoin, 5-methyl-	-0.518085770898941	-0.308779061793579	-0.076817176306518
Streptococcaceae	X6092	3,4-dimethyl-5-carboxyethyl-2-furanpentanoic acid	-0.503157626031892	-0.308533992255031	-0.091528221484069
Alphaproteobacteria_(unclassified_order)	X177	O-glutarylcarnitine;Glutarylcarnitine	-0.536162083783407	-0.306358927735448	-0.056350996297909
Rhodospirillaceae	X7233	Pentadecane, n-	-0.494876860821673	-0.305097657366806	-0.097752365015198
Synergistaceae	X7207	Maltotriose	-0.513711526331251	-0.304310742025575	-0.056429938957126
Bacteroidetes_(unclassified_class)	X153	Withaperuvin B	-0.510818528009142	-0.302942794830308	-0.087534151572392
Lactobacillaceae	X32	(-)-Jolkinol B	-0.507285660235403	-0.302672935448592	-0.066362078996977
Synergistaceae	X369	PE-Cer(d15:2(4E,6E)/22:0(2OH))	-0.520039729419392	-0.301910227009001	-0.075322151580024
Bacteroidales_(unclassified_family)	X331	cholesterol sulfate	0.05495088640436	0.303038277691988	0.518790647327614
Bifidobacteriaceae	X7145	PI-Cer(t20:0/22:0(2OH))	0.094820634742746	0.306938185585807	0.497725171587029
Coriobacteriaceae	X36	PG(16:1(9Z)/22:4(7Z,10Z,13Z,16Z))	0.090319549658451	0.307008813702457	0.4939851224223
Lactobacillaceae	X7038	PE(20:2(11Z,14Z)/22:5(4Z,7Z,10Z,13Z,16Z))	0.082316717589988	0.307384620569576	0.509621581090231
Mollicutes_(unclassified_order)	X406	SM(d16:1/22:0)	0.053138416615765	0.310727569029368	0.538780896013864
Mollicutes_(unclassified_order)	X7003	MGDG(20:5(5Z,8Z,11Z,14Z,17Z)/18:3(9Z,12Z,15Z))	0.045908974683232	0.311116870361986	0.545884279931508
Desulfovibrionales_(unclassified_family)	X499	3-Deoxyvitamin D3	0.057227768634548	0.31350170053877	0.533984327626467
Mollicutes_(unclassified_order)	X362	PC(22:4(7Z,10Z,13Z,16Z)/0:0)	0.067267156787301	0.315515698003986	0.543182734048356
Victivallaceae	X6619	FAHFA(18:1(9Z)/13-O-18:0)	0.0981300890361	0.315962541220477	0.514637106256656
Mollicutes_(unclassified_order)	X420	PC(16:1(9Z)/0:0);PC(16:1(9E)/0:0)	0.06789006796849	0.323385097332867	0.54483050743202
Alphaproteobacteria_(unclassified_order)	X327	Anandamide (20:5, n-3)	0.089101474553077	0.325595790874115	0.544535648623506
Mollicutes_(unclassified_order)	X500	Butyrylcarnitine;-	0.089747368553412	0.337485121550267	0.561375046093987
Mollicutes_(unclassified_order)	X331	cholesterol sulfate	0.092348216747257	0.337636542617832	0.559376024972936
Verrucomicrobiaceae	X6564	PS(19:0/0:0)	0.128759279254497	0.341341472211479	0.529676112527731
Clostridia_(unclassified_order)	X7241	Aniline, 3,4-dimethyl-	0.116249361338189	0.344579919351646	0.543457936346808
Coriobacteriaceae	X75	N-stearoyl tyrosine	0.124515361721117	0.347917809060995	0.539677034338611
Pasteurellaceae	X6860	PE(16:0/P-18:1(11Z))	0.110626867338321	0.352072533033137	0.561974288810277
Mollicutes_(unclassified_order)	X458	20:2-Glc-Campesterol	0.09268739604301	0.354270727175613	0.583097044793681
Victivallaceae	X6092	3,4-dimethyl-5-carboxyethyl-2-furanpentanoic acid	0.149465970373997	0.357982005514131	0.544108258769192
Bacteroidales_(unclassified_family)	X6564	PS(19:0/0:0)	0.11282394544823	0.362923275679255	0.576605146234015
Erysipelotrichaceae	X7253	Proline	0.161608788825078	0.371488769580704	0.564777128255811
Mollicutes_(unclassified_order)	X32	(-)-Jolkinol B	0.114417817538072	0.371814291247775	0.589631720677055

NOTE: Unclassified taxa may contain more than one taxon together in the same unclassified "bin". They are presented here just for the purpose of showing the raw results from the analysis pipeline.

Supplementary table 4

Family	Metabolite Peak ID	Metabolite MSI 3 ID	p2.5	mean	p97.5
Rhodospirillaceae	X490	PA(O-20:0/13:0)	-0.680142934001683	-0.524624064447755	-0.330654412256203
Rhodospirillaceae	X458	20:2-Glc-Campesterol	-0.652329355206323	-0.472379321272196	-0.269145261161573
Rhodospirillaceae	X418	Sorbitan stearate	-0.642089375043526	-0.467592359726838	-0.267432518504219
Rhodospirillaceae	X331	cholesterol sulfate	-0.637522879054015	-0.467385415930342	-0.260710471192778
Rhodospirillaceae	X70	(3S,5R,6S,7E,9x)-7-Megastigmene-3,6,9-triol 9-glucoside	-0.63175863911792	-0.457296605681074	-0.248751878786233
Rhodospirillaceae	X313	iodovulone I	-0.621926868760351	-0.433052718380625	-0.209838866191914
Rhodospirillaceae	X5	iodovulone I	-0.580434365092728	-0.402252531717173	-0.19802549486951
Alphaproteobacteria_(unclassified_order)	X263	Epigallocatechin 3-O-caffeate	-0.590667684144676	-0.396473070519331	-0.18129476776034
Desulfovibrionales_(unclassified_family)	X32	(-)-Jolkinol B	-0.606863640086231	-0.385353395364754	-0.114958566103109
Rhodospirillaceae	X276	SM(d18:0/24:0)	-0.579372198061467	-0.384334785888299	-0.167523157739087
Rhodospirillaceae	X500	Butyrylcarnitine;-	-0.579264532381554	-0.383593252874203	-0.155582913295155
Rhodospirillaceae	X420	PC(16:1(9Z)/0:0);PC(16:1(9E)/0:0)	-0.573217484960804	-0.371313455868055	-0.145846417788403
Rhodospirillaceae	X32	(-)-Jolkinol B	-0.564254219775721	-0.366473991216446	-0.147408613595834
Coriobacteriaceae	X7246	Proline, 4-hydroxy-, trans-	-0.545845043400712	-0.364149341782953	-0.155460436164511
Rhodospirillaceae	X327	Anandamide (20:5, n-3)	-0.5589656134402	-0.363433063106752	-0.137216889461433
Coriobacteriaceae	X348	Fuconic acid	-0.542821660884326	-0.358726610908622	-0.14777679079113
Eubacteriaceae	X7285	Estrone, 16alpha-hydroxy-	-0.563105629950444	-0.358059783835206	-0.124600540118623
Sutterellaceae	X7160	Dodecane	-0.542894096350498	-0.357195371838911	-0.16095602769994
Clostridia_(unclassified_order)	X6808	OKHdiA-PS	-0.555229549909318	-0.35408606856315	-0.130375695754198
Rhodospirillaceae	X144	1alpha,24,25,28-tetrahydroxyergocalciferol	-0.554805127952955	-0.34384266063121	-0.112517566821896
Coriobacteriaceae	X276	SM(d18:0/24:0)	-0.528211890302272	-0.340732350520166	-0.124720602691767
Rhodospirillaceae	X399	PE(O-20:0/22:6(4Z,7Z,10Z,13Z,16Z,19Z))	-0.54666658096255	-0.337743323236938	-0.117646781265978
Anaeroplasmataceae	X7183	Alanine, beta-	-0.527443092910299	-0.337517716217262	-0.106342737535808
Rhodospirillaceae	X502	PE(P-18:0/20:5(5Z,8Z,11Z,14Z,17Z))	-0.536535943110814	-0.336794365554982	-0.121833639874645
Coriobacteriaceae	X429	PC(16:0/18:1(6Z));PC(16:0/18:1(6E))	-0.529833883453034	-0.336157400502641	-0.124281864728455
Clostridia_(unclassified_order)	X32	(-)-Jolkinol B	-0.53518749220878	-0.333863477240523	-0.112657884290361
Rhodospirillaceae	X406	SM(d16:1/22:0)	-0.539834688497207	-0.331603777857124	-0.0978548123088663
Rhodospirillaceae	X497	SM(d17:1/24:1)	-0.538322924407134	-0.331386785830371	-0.104356886545887
Peptococcaceae_1	X7176	3-Methyl-2-oxopentanoic-acid	-0.513162541568671	-0.321360962205722	-0.0891985004036077
Rhodospirillaceae	X362	PC(22:4(7Z,10Z,13Z,16Z)/0:0)	-0.529580824950569	-0.320349296210807	-0.0826741069391441
Rhodospirillaceae	X287	Fuconic acid	-0.519608937431131	-0.319831189404036	-0.0803170998199828
Rhodospirillaceae	X498	SM(d18:2/21:0)	-0.518700066379461	-0.318225048215602	-0.0900090529434954
Desulfovibrionales_(unclassified_family)	X490	PA(O-20:0/13:0)	-0.550736908080095	-0.318016667684039	-0.0596618159653456
Firmicutes_(unclassified_class)	X6176	3,3-Dibromo-2-n-hexylacrylic acid	-0.509964005903051	-0.317690322228108	-0.0945552350164938
Bifidobacteriaceae	X7163	2-amino-2-deoxy-glucose	-0.512672539154187	-0.317133115689661	-0.104893025689904

Rhodospirillaceae	X6176	3,3-Dibromo-2-n-hexylacrylic acid	-0.52942298411052	-0.315868131354318	-0.0702246934560463
Desulfovibrionales_(unclassified_family)	X498	SM(d18:2/21:0)	-0.543216427606338	-0.315708542840385	-0.0512334564514758
Alphaproteobacteria_(unclassified_order)	X474	Acevaltrate	-0.534198419622907	-0.314982263575475	-0.0715204436948824
Desulfovibrionales_(unclassified_family)	X500	Butyrylcarnitine;-	-0.560234024925327	-0.314212849130211	-0.0498298798187397
Veillonellaceae	X499	3-Deoxyvitamin D3	-0.516649242275483	-0.311965425762042	-0.0979730788437419
Lactobacillaceae	X7183	Alanine, beta-	-0.507931964689189	-0.306176938972076	-0.0822381582378314
Anaeroplasmataceae	X115	Hydroxybutyrylcarnitine;3-hydroxybutyrylcarnitine	-0.522746749124573	-0.305478005686273	-0.070512711163275
Desulfovibrionales_(unclassified_family)	X331	cholesterol sulfate	-0.540769709325233	-0.303420611916986	-0.0364575603091914
Anaeroplasmataceae	X70	(3S,5R,6S,7E,9x)-7-Megastigmene-3,6,9-triol 9-glucoside	-0.515644116525647	-0.301903782784798	-0.0628946022292624
Clostridia_(unclassified_order)	X490	PA(O-20:0/13:0)	-0.506986999580229	-0.301770522101974	-0.0691256070351358
Veillonellaceae	X7163	2-amino-2-deoxy-glucose	-0.498713669600236	-0.301087867694876	-0.0810885951938005
Alphaproteobacteria_(unclassified_order)	X191	Sphinganine-phosphate	-0.510008416139907	-0.300360982610848	-0.0563805796456758
Desulfovibrionales_(unclassified_family)	X7191	Methionine	0.0184752285653881	0.300047759638269	0.538022862402669
Rhodospirillaceae	X439	PI(16:0/20:1(11Z))	0.0662032399200347	0.30049510610299	0.522690943011633
Desulfovibrionales_(unclassified_family)	X264	6-Keto-decanoylcarnitine	0.0457388436443856	0.303319803834669	0.532494717308614
Clostridia_(unclassified_order)	X7232	Norvaline, DL-	0.0489015113110592	0.303728617284754	0.557338463078944
Mollicutes_(unclassified_order)	X369	PE-Cer(d15:2(4E,6E)/22:0(2OH))	0.0505214821834933	0.306278220992479	0.544271462139193
Desulfovibrionales_(unclassified_family)	X153	Withaperuvin B	0.0299444948489795	0.306356130590442	0.543916164584856
Clostridia_(unclassified_order)	X7191	Methionine	0.0771739525792996	0.30639366744508	0.511611078792874
Clostridia_(unclassified_order)	X170	PA(P-18:0/17:2(9Z,12Z))	0.0762322638977738	0.306729675539917	0.507769861562481
Victivallaceae	X7164	Glucose, 2-amino-2-deoxy-	0.0667549011914025	0.309019859938056	0.519533365353118
Rhodospirillaceae	X7287	Tartronic acid	0.0640867927548175	0.309040396149383	0.524712302711954
Bacteria_(unclassified_phylum)	X7232	Norvaline, DL-	0.0798443245652232	0.309547072540667	0.512776867736379
Desulfovibrionales_(unclassified_family)	X439	PI(16:0/20:1(11Z))	0.0520157139367747	0.309920154864014	0.542652914980445
Firmicutes_(unclassified_class)	X7164	Glucose, 2-amino-2-deoxy-	0.0870588948856106	0.311906725992879	0.509124970059048
Veillonellaceae	X6277	Veranisatin C	0.0914107387659148	0.312710017729822	0.520427831963658
Rhodospirillaceae	X511	PC(18:3(9Z,12Z,15Z)/0:0)	0.0759769914798798	0.316725198388488	0.513166329231808
Deltaproteobacteria_(unclassified_order)	X463	PC(P-16:0/20:3(8Z,11Z,14Z))	0.0764367136860735	0.318601464832989	0.533696439874892
Firmicutes_(unclassified_class)	X7160	Dodecane	0.112972074864398	0.322691527455383	0.516412965620127
Mollicutes_(unclassified_order)	X348	Fuconic acid	0.0608015549464156	0.323741981912049	0.54858042784979
Rhodospirillaceae	X243	PS(20:3(8Z,11Z,14Z)/0:0)	0.0787159010326153	0.32516120494507	0.543338174647604
Enterobacteriaceae	X485	SM(d18:1/21:0)	0.113958650774568	0.327997388011809	0.528069293676783
Enterobacteriaceae	X430	PC(P-20:0/18:3(6Z,9Z,12Z))	0.12916018258341	0.329929736474712	0.532474878859538
Clostridiaceae_1	X7276	Galactose, 2-amino-2-deoxy-, D-	0.109490818569758	0.330623282423481	0.522487082400557
Mollicutes_(unclassified_order)	X70	(3S,5R,6S,7E,9x)-7-Megastigmene-3,6,9-triol 9-glucoside	0.0666799868403681	0.333908405903696	0.561822779016637
Bacteria_(unclassified_phylum)	X7281	Benzaldehyde	0.114704034310367	0.335536553616951	0.527132242330473
Mollicutes_(unclassified_order)	X429	PC(16:0/18:1(6Z));PC(16:0/18:1(6E))	0.0967396635506875	0.344877935515207	0.570078812218249
Streptococcaceae	X6984	PE(20:4(8Z,11Z,14Z,17Z)/20:4(8Z,11Z,14Z,17Z))	0.146716234707441	0.350175426092395	0.536238530096667
Mollicutes_(unclassified_order)	X130	7,3'-Dihydroxy-4'-methoxy-8-methylflavan	0.108269811998038	0.353773947852656	0.585336048554741

Bacteroidetes_(unclassified_class)	X191	Sphinganine-phosphate	0.109102253673469	0.354394808318456	0.571932736707865
Mollicutes_(unclassified_order)	X276	SM(d18:0/24:0)	0.11022340412263	0.364275331717495	0.588365036250029
Mollicutes_(unclassified_order)	X313	iodovulone I	0.120639688356732	0.364912614687025	0.581566003219928
Lactobacillaceae	X7081	PS(19:0/22:6(4Z,7Z,10Z,13Z,16Z,19Z))	0.122264337596651	0.372469772720566	0.579508460733243
Rhodospirillaceae	X7164	Glucose, 2-amino-2-deoxy-	0.150847693595668	0.389083917399679	0.592042518165813
Anaeroplasmataceae	X363	3-carboxy-4-methyl-5-pentyl-2-furanpropanoic acid	0.163662083482761	0.394814810537893	0.60007167556639
Mollicutes_(unclassified_order)	X443	GlcCer(d18:1(8Z)/21:0(2OH[R]));GlcCer(d18:1(8E)/21:0(2OH[R]))	0.162058861340323	0.3996175099502	0.610364990793029
Mollicutes_(unclassified_order)	X115	Hydroxybutyrylcarnitine;3-hydroxybutyrylcarnitine	0.164026786660655	0.402266823582697	0.618147700778687
Rhodospirillaceae	X7191	Methionine	0.189991511673198	0.415105155919161	0.607762446829899
Rhodospirillaceae	X7160	Dodecane	0.193001069852151	0.421089126160165	0.618976674423706
Anaeroplasmataceae	X511	PC(18:3(9Z,12Z,15Z)/0:0)	0.21139554138767	0.441381588932417	0.633682309935578

NOTE: Unclassified taxa may contain more than one taxon together in the same unclassified "bin". They are presented here just for the purpose of showing the raw results from the analysis pipeline.

Supplementary Table 5

Phylum	Metabolite Peak ID	Metabolite MSI 3 ID	p2.5	Correlation	p97.5
Lentisphaerae	X7206	Methionine, N-formyl-	-0.576027419896728	-0.382574541214548	-0.169927945973091
Verrucomicrobia	X7038	PE(20:2(11Z,14Z)/22:5(4Z,7Z,10Z,13Z,16Z))	-0.53064322973377	-0.341383047394019	-0.123136630343093
Lentisphaerae	X7171	Pyroglutamic acid	-0.517570532185033	-0.333351831855983	-0.113676749919875
Bacteria_(unclassified_phylum)	X6277	Veranisatin C	-0.513576068926666	-0.315227720280151	-0.096991441092894
Lentisphaerae	X430	PC(P-20:0/18:3(6Z,9Z,12Z))	-0.50647858863548	-0.315064007654969	-0.102483009080933
Synergistetes	X7253	Proline	-0.530633113660822	-0.314842680540688	-0.085969228487273
Synergistetes	X7207	Maltotriose	-0.52096395230243	-0.312128079225767	-0.073373744999617
Synergistetes	X348	Fuconic acid	-0.524431746877642	-0.312108147768935	-0.072706300947173
Lentisphaerae	X243	PS(20:3(8Z,11Z,14Z)/0:0)	-0.508012135860675	-0.311936605793749	-0.088979848542703
Synergistetes	X7160	Dodecane	-0.527946586689352	-0.310044352854186	-0.069968157127773
Synergistetes	X7175	Dodecane	-0.523546980330141	-0.308028926896016	-0.068399959956145
Lentisphaerae	X7038	PE(20:2(11Z,14Z)/22:5(4Z,7Z,10Z,13Z,16Z))	-0.511464429841719	-0.306980096148593	-0.079970353920536
Tenericutes	X7206	Methionine, N-formyl-	0.057274525994282	0.30389329065881	0.529628587999238
Lentisphaerae	X7081	PS(19:0/22:6(4Z,7Z,10Z,13Z,16Z,19Z))	0.075724200729519	0.30943168679695	0.512670237929039
Verrucomicrobia	X6564	PS(19:0/0:0)	0.106357022295602	0.334514913975828	0.530192335504182
Lentisphaerae	X6619	FAHFA(18:1(9Z)/13-O-18:0)	0.14639071853481	0.345822430149375	0.542210733181181
Lentisphaerae	X6092	3,4-dimethyl-5-carboxyethyl-2-furanpentanoic acid	0.182698481135648	0.400946130179642	0.594199216918368

NOTE: Unclassified taxa may contain more than one taxon together in the same unclassified "bin". They are presented here just for the purpose of showing the raw results from the analysis pipeline.

Supplementary Table 6

Phylum	Metabolite Peak ID	Metabolite MSI 3 ID	p2.5	mean	p97.5
Lentisphaerae	X9	OHOHA-PS	0.069738532604757	0.300944512077806	0.510569271117612
Tenericutes	X7081	PS(19:0/22:6(4Z,7Z,10Z,13Z,16Z, 19Z))	0.072302912133793	0.303950256886272	0.512983791312212
Lentisphaerae	X7164	Glucose, 2-amino-2-deoxy-	0.067759011777312	0.30720031042381	0.515732093283264
Lentisphaerae	X463	PC(P-16:0/20:3(8Z,11Z,14Z))	0.096769689687906	0.313586727332336	0.519477064875953
Bacteria_(unclassified_phylum)	X7281	Benzaldehyde	0.103772998543508	0.33102607902869	0.535921986050809
Bacteria_(unclassified_phylum)	X7232	Norvaline, DL-	0.121800087764099	0.350808076361992	0.548477051850195

NOTE: Unclassified taxa may contain more than one taxon together in the same unclassified "bin". They are presented here just for the purpose of showing the raw results from the analysis pipeline.

Supplementary table 7 (MODELS)

Model idx	Model description	Independent variable	Dependent variable	Adjusted for	Statistical measurement		Change after feature selection (if attempted)		Validation method
					**PLS-DA Correct classification rates**		**PLS-DA Correct classification rates**		
**1**	Metabolomics data for disease status	Metabolite features from GC-MS analysis	Disease status (PD/Control)	none	81 %		n/a		60:40 train:test split with 100 resampling
**2**	Metabolomics data for disease status	Metabolite features from LC-MS positive mode analysis	Disease status (PD/Control)	none	77 %				60:40 train:test split with 100 resampling
**3**	Metabolomics data for disease status	Metabolite features from LC-MS negative mode analysis	Disease status (PD/Control)	none	72 %				60:40 train:test split with 100 resampling
					**SVM Correct classification rates**		**SVM Correct classification rates**		
					7585 metabolite features		*Top 759 metabolite features*		
4	Metabolomics data for disease status	Metabolite features combined	Disease status (PD/Control)	none	82 %		85 %		60:40 train:test split with 100 resampling
5	Metabolomics data for disease status	Metabolite features combined	Disease status (PD/Control)	all with hypecholesterolaemia	82 %		82 %		60:40 train:test split with 100 resampling
6	Metabolomics data for disease status in male only	Metabolite features combined	Disease status (PD/Control)	all males	78 %		78 %		60:40 train:test split with 100 resampling
7	Metabolomics data for disease status in female only	Metabolite features combined	Disease status (PD/Control)	all females	75 %		73 %		60:40 train:test split with 100 resampling

8	Metabolomics data for disease status while looking for any effects or associations with age, BMI, clinical scores and dietary intake data	Metabolite features combined	Disease status (PD/Control)	BMI, Stool_diary_D1_defecations_per_week, Stool_diary_D1_characteristic_average, Weight_g, vyotaro_cm, age_at_stool_collection, days_between_samples, GDS15, MMSE_total, NMSQuest_total, NMSS_total, RBDSQ, Rome_III_constip_defec_sumscore_9.15, SDQ_total, sniffinsticks, tobacco_when_last, Wexner_total, Energy_Kj, Energy_kc, Ethanol_g, Fat_g, Carbs_g, Sugar_g, Sucrose_g, Protein_g, Salt_g, Safa_g, Mufa_g, Pufa_g, Trans_g, EPA_mg, DHA_mg, Cholesterol, Sterols, Organicacids_g, Starch_g, Fiber_g, Fiber_soluble_g, Fiber_insoluble_g, Fructose_g, Maltose_g, Lactose_g, Glucose_g, Galactose_g, Calcium, Iron, Iodine, Potassium, Magnesium, Sodium, Phosphorus, Selenium, Zinc, Folate, Niacin, Pyridoxin, Riboflavin, Thiamine, VitaminB12, VitaminC, VitaminA, Carotenoids, VitaminD, VitaminE, VitaminK, Dairy, Cheese, Probiotics, Cereal, Meat, Fish, Plants, Vegetables, Fruits, Berries, Fungi, Nuts, Candy, Coffee, Tea, butyric_acid, acetic_acid, isobutyric_acid, propionic_acid.	84 %		84 %		60:40 train:test split with 100 resampling
					**PLS_R^2**	**PLS_RMSE**	**PLS_R ^2**	**PLS_RMSE**	**Validation method**
					7585 metabolite features		*Top 759 metabolite features*		
9	Metabolomics data association with PD clinical criteria (within-PD)	Metabolite features combined	GDS15	meds_SSRI, age at sampling, time since motor onset	-1,318	5,422	-0,321	4,093	60:40 train:test split with 100 resampling
10	Metabolomics data association with PD clinical criteria (within-PD)	Metabolite features combined	MMSE_total	meds_antichlonergic, meds_tricyclics, age at sampling, time since motor onset	-1,048	2,235	-0,245	1,743	60:40 train:test split with 100 resampling
11	Metabolomics data association with PD clinical criteria (within-PD)	Metabolite features combined	NMSS_total	age at sampling, time since motor onset	-2,959	72,231	-0,045	37,117	60:40 train:test split with 100 resampling

12	Metabolomics data association with PD clinical criteria (within-PD)	Metabolite features combined	RBDSQ	none	-2,863	5,877	-0,449	3,6	60:40 train:test split with 100 resampling
13	Metabolomics data association with PD clinical criteria (within-PD)	Metabolite features combined	Rome_III_constip_defec_sumscore_9.15	meds_antichlonergic, meds_tricyclics, meds_opioids, meds_constipation, fiber_g, age at sampling, time since motor onset	-4,935	12,696	-0,155	5,602	60:40 train:test split with 100 resampling
14	Metabolomics data association with PD clinical criteria (within-PD)	Metabolite features combined	SCS_PD_total	meds_antichlonergic, meds_tricyclics, age at sampling, time since motor onset, LED	-1,649	6,871	-0,089	4,406	60:40 train:test split with 100 resampling
15	Metabolomics data association with PD clinical criteria (within-PD)	Metabolite features combined	SDQ_total	age at sampling, time since motor onset, LED	-3,307	10,01	-0,364	5,634	60:40 train:test split with 100 resampling
16	Metabolomics data association with PD clinical criteria (within-PD)	Metabolite features combined	UPDRS_V_OFF	age at sampling, time since motor onset, LED	-2,411	1,365	-0,125	0,784	60:40 train:test split with 100 resampling
17	Metabolomics data association with PD clinical criteria (within-PD)	Metabolite features combined	UPDRS_II_total	age at sampling, time since motor onset, LED	-1,596	8,609	-0,021	5,399	60:40 train:test split with 100 resampling
18	Metabolomics data association with PD clinical criteria (within-PD)	Metabolite features combined	UPDRS_III_total_OFF	age at sampling, time since motor onset, LED, beta blockers	-1,089	16,047	0,186	10,02	60:40 train:test split with 100 resampling
19	Metabolomics data association with PD clinical criteria (within-PD)	Metabolite features combined	Wexner_total	meds_antichlonergic, meds_tricyclics, meds_opioids, meds_constipation, fiber_g, age at sampling, time since motor onset	-2,542	8,653	-0,014	4,63	60:40 train:test split with 100 resampling
					Key 139 metabolite features		*Top 14 metabolites features*		**Validation method**
20	Effect of PD drug dosage on key metabolomic features	Metabolite features combined	meds_entacapone_mg	Age at stool collection, time from motor onset	-0,553	311,377	-3,303	518,319	LOOCV

21	Effect of PD drug dosage on key metabolomic features	Metabolite features combined	meds_levodopa_depot_mg	Age at stool collection, time from motor onset	-1,858	309,845	-1,126	267,262	LOOCV
22	Effect of PD drug dosage on key metabolomic features	Metabolite features combined	meds_levodopa_entacapone_mg	Age at stool collection, time from motor onset	-0,604	267,694	0,468	154,225	LOOCV
23	Effect of PD drug dosage on key metabolomic features	Metabolite features combined	meds_levodopa_IR_mg	Age at stool collection, time from motor onset	-29,299	784,168	-1,992	246,407	LOOCV
24	Effect of PD drug dosage on key metabolomic features	Metabolite features combined	meds_pramipexole_mg	Age at stool collection, time from motor onset	-0,445	0,96	-1,463	1,571	LOOCV
25	Effect of PD drug dosage on key metabolomic features	Metabolite features combined	meds_ropinirole_mg	Age at stool collection, time from motor onset	-95,266	61,774	-5,158	15,623	LOOCV
26	Effect of PD drug dosage on key metabolomic features	Metabolite features combined	UPDRS_III_total_OFF	Age at stool collection, time from motor onset	-2,325	19,216	-0,283	11,936	LOOCV
27	Effect of PD drug dosage on key metabolomic features	Metabolite features combined	calculated_time_from_motor_onset	Age at stool collection, UPDRSIII	-3,301	8,061	-0,385	4,574	LOOCV
					**Logistic regression classification accuracy**		**Logistic regression classification accuracy**		**Validation method**
					7585 metabolite features		*Top 759 metabolite features*		
28	Metabolomics data association with PD clinical criteria (within-PD)	Metabolite features combined	Progression_CAT	none	64 %		68 %		60:40 train:test split with 100 resampling
29	Metabolomics data association with PD clinical criteria (within-PD)	Metabolite features combined	Rome_III_IBS_criteria_fulfilled	meds_antichlonergic, meds_tricyclics, meds_opioids, meds_constipation, fiber_g, age at sampling, time since motor onset	60 %		69 %		60:40 train:test split with 100 resampling
					Key 139 metabolite features		*Top 14 metabolite features*		
30	Effect of PD drug intake on key metabolomic features	Metabolite features combined	meds_COMT_inhibitor	Age at stool collection, time from motor onset	63 %		54 %		LOOCV
31	Effect of PD drug intake on key metabolomic features	Metabolite features combined	meds_dopa	Age at stool collection, time from motor onset	33 %		53 %		LOOCV

32	Effect of PD drug intake on key metabolomic features	Metabolite features combined	meds_dopamine_agonist	Age at stool collection, time from motor onset	33 %		50 %			LOOCV
33	Effect of PD drug intake on key metabolomic features	Metabolite features combined	meds_MAO_inhibitor	Age at stool collection, time from motor onset	32 %		53 %			LOOCV
34	Effect of PD drug intake on key metabolomic features	Metabolite features combined	meds_rasagiline_mg	Age at stool collection, time from motor onset	56 %		64 %			LOOCV
35	Effect of PD drug intake on key metabolomic features	Metabolite features combined	meds_selegeline_mg	Age at stool collection, time from motor onset	34 %		62 %			LOOCV

Supplementary Figure - Unknown 1 spectra

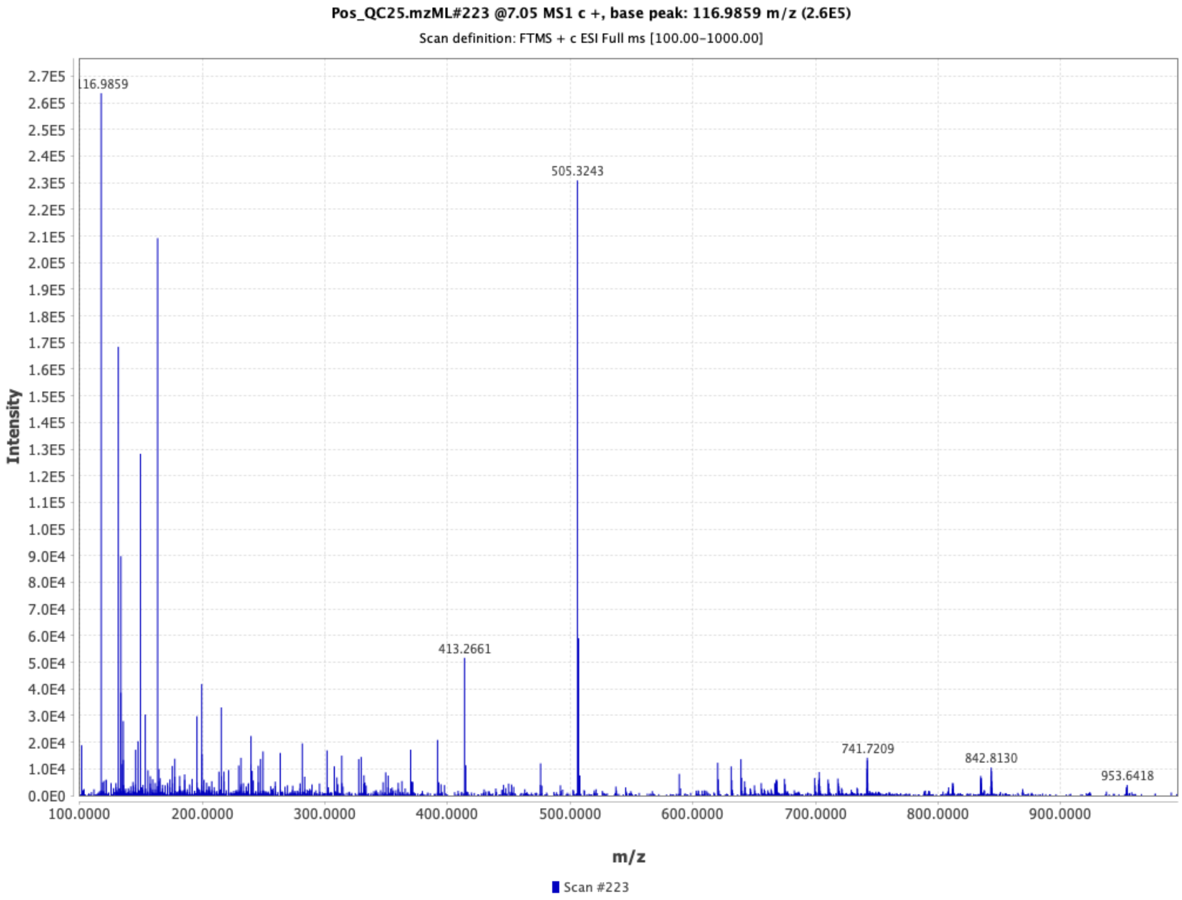

Supplementary Figure - Unknown 2 spectra

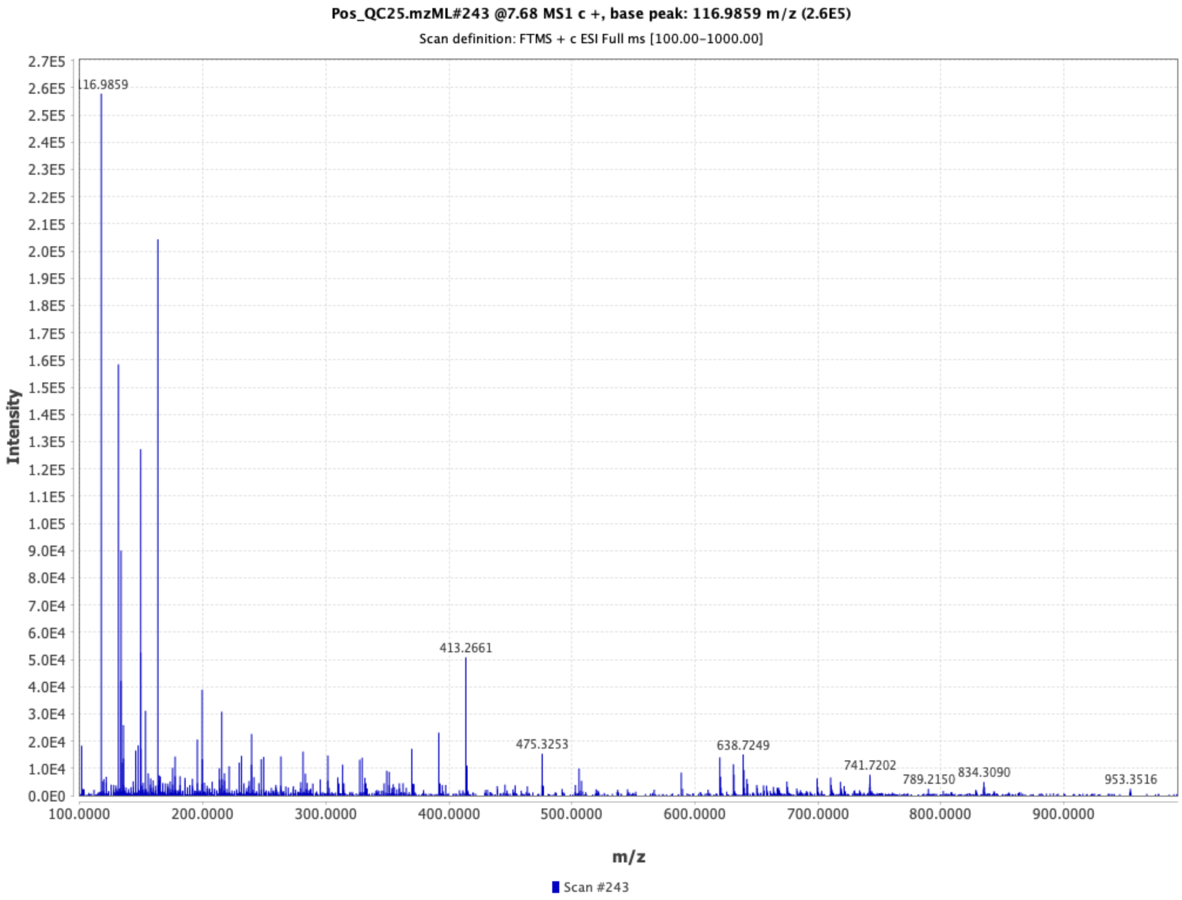

Supplementary Figure - Unknown 3 spectra

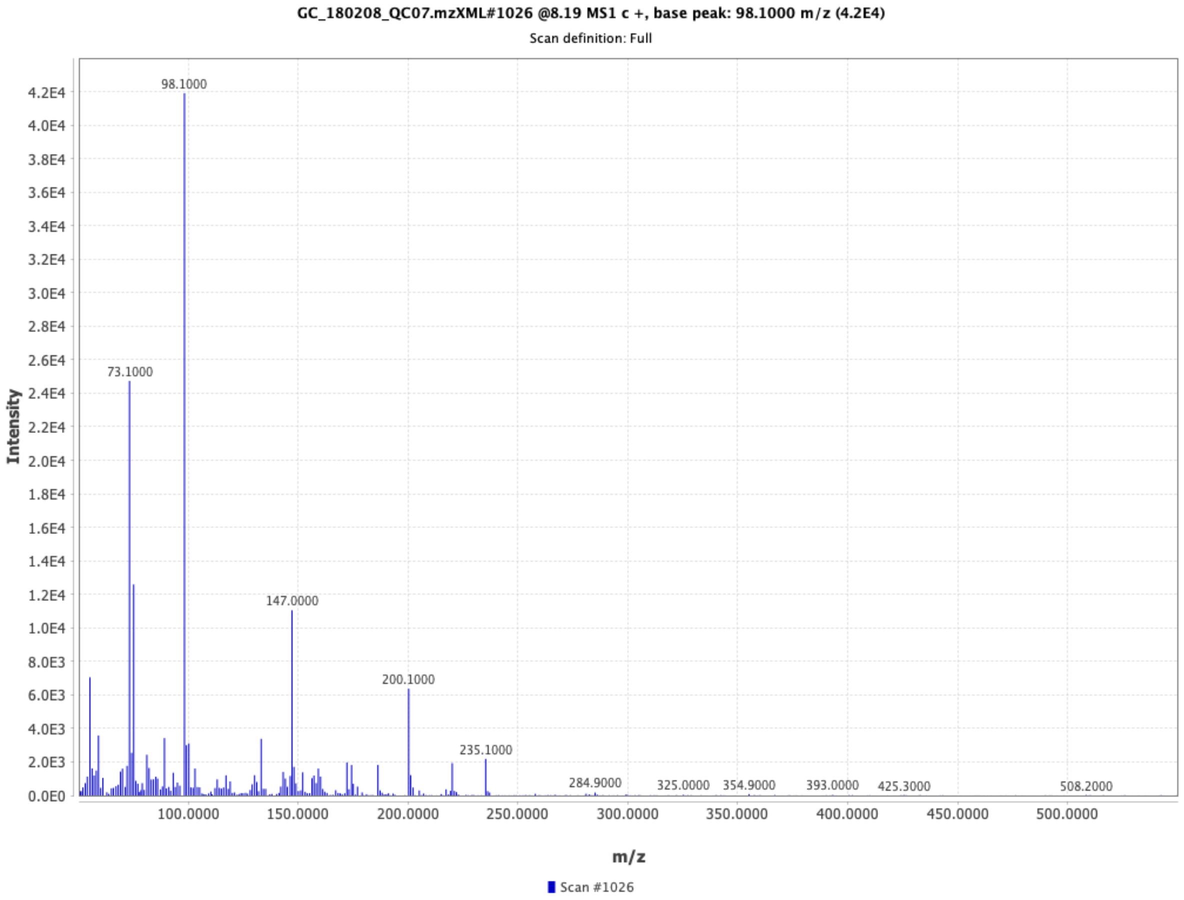

Supplementary Figure - Unknown 4 spectra

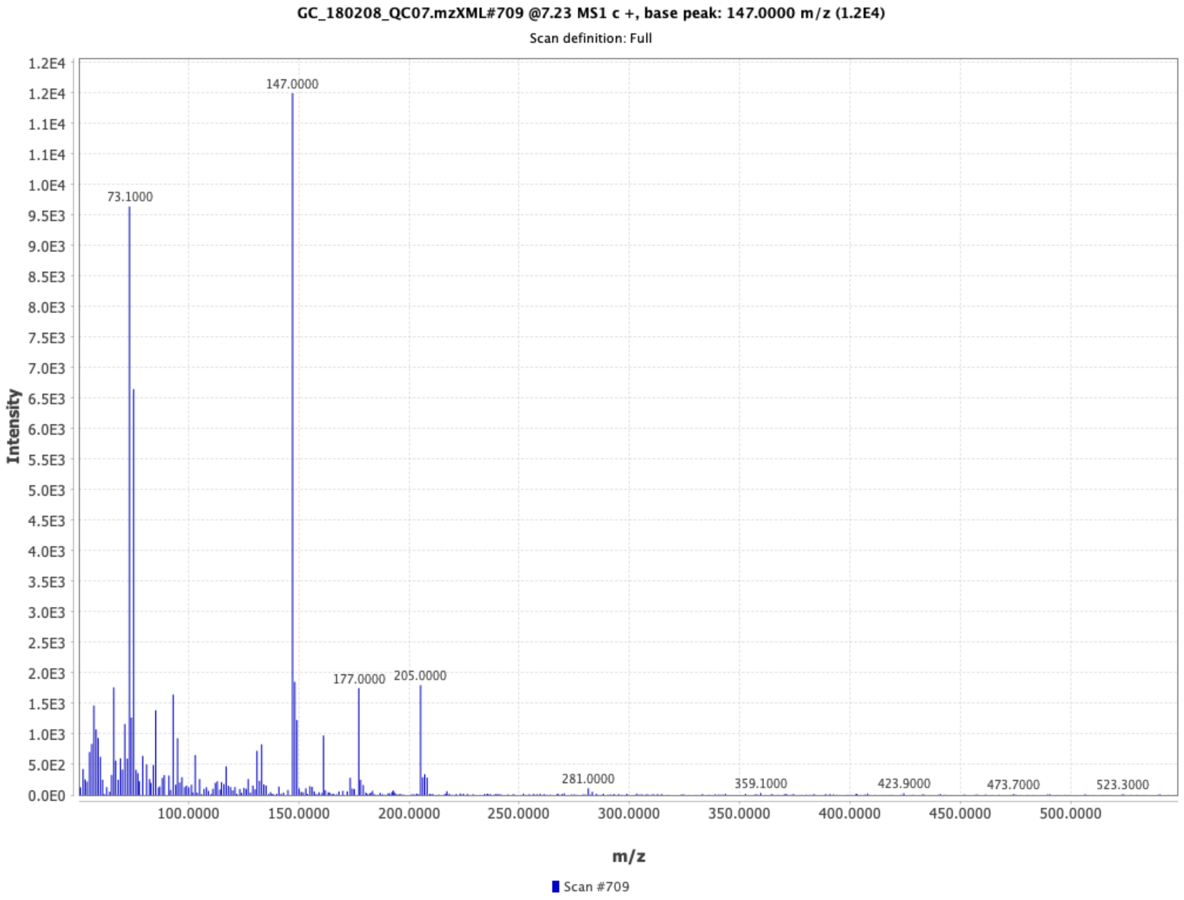

Supplement: Supplementary file 2 — Supplementary file [file 41531_2022_300_MOESM2_ESM.pdf]
